# Addressing the quality and scope of paediatric primary care in South Africa: evaluating contextual impacts of the introduction of the Practical Approach to Care Kit for children (PACK Child)

**DOI:** 10.1186/s12913-020-05201-w

**Published:** 2020-05-29

**Authors:** Jamie Murdoch, Robyn Curran, Ruth Cornick, Sandy Picken, Max Bachmann, Eric Bateman, Makhosazana Lungile Simelane, Lara Fairall

**Affiliations:** 1 School of Health Sciences, University of East Anglia, Edith Cavell Building, Colney Lane, Norwich, NR4 7TJ UK; 2 Knowledge Translation Unit, University of Cape Town Lung Institute, University of Cape Town, Mowbray, 7700 South Africa; 3 Department of Medicine, University of Cape Town, Observatory, 7925, South Africa; 4 Norwich Medical School, University of East Anglia, Norwich, NR4 7TJ UK; 5 King’s Global Health Institute, King’s College London, London, SE1 9NH UK

**Keywords:** Child health, Health systems evaluation, Paediatrics, Prevention strategies, Other study design

## Abstract

**Background:**

Despite significant reductions in mortality, preventable and treatable conditions remain leading causes of death and illness in children in South Africa. The PACK Child intervention, comprising clinical decision support tool (guide), training strategy and health systems strengthening components, was developed to expand on WHO’s Integrated Management of Childhood Illness programme, extending care of children under 5 years to those aged 0–13 years, those with chronic conditions needing regular follow-up, integration of curative and preventive measures and routine care of the well child. In 2017–2018, PACK Child was piloted in 10 primary healthcare facilities in the Western Cape Province. Here we report findings from an investigation into the contextual features of South African primary care that shaped how clinicians delivered the PACK Child intervention within clinical consultations.

**Methods:**

Process evaluation using linguistic ethnographic methodology which provides analytical tools for investigating human behaviour, and the shifting meaning of talk and text within context. Methods included semi-structured interviews, focus groups, ethnographic observation, audio-recorded consultations and documentary analysis. Analysis focused on how mapped contextual features structured clinician-caregiver interactions.

**Results:**

Primary healthcare facilities demonstrated an institutionalised orientation to minimising risk upheld by provincial documentation, providing curative episodic care to children presenting with acute symptoms, and preventive care including immunisations, feeding and growth monitoring, all in children 5 years or younger. Children with chronic illnesses such as asthma rarely receive routine care. These contextual features constrained the ability of clinicians to use the PACK Child guide to facilitate diagnosis of long-term conditions, elicit and manage psychosocial issues, and navigate use of the guide alongside provincial documentation.

**Conclusion:**

Our findings provide evidence that PACK Child is catalysing a transition to an approach that strikes a balance between assessing and minimising risk on the day of acute presentation and a larger remit of care for children over time. However, optimising success of the intervention requires reviewing priorities for paediatric care which will facilitate enhanced skills, knowledge and deployment of clinical staff to better address acute illnesses and long-term health conditions of children of all ages, as well as complex psychosocial issues surrounding the child.

## Background

The three principal objectives of the 2016–2030 Global Strategy for Women’s, Children’s and Adolescents’ Health are Survive, Thrive and Transform, including the need to build resilience in health systems, improve the quality of health services and equity in their coverage [1]. These objectives align with the United Nation’s Sustainable Development Goals [2], which envisage the highest standards of physical and mental well-being for these vulnerable groups. However, large inequalities persist in access to, and the quality of care in many low and middle-income countries (LMICs) [3]. In South Africa, the management of common childhood illnesses at a primary healthcare level remains poor with preventable and treatable conditions, particularly pneumonia and diarrhoea, remaining the leading causes of death in children under five [4]. With under-five mortality rate of 42 per 1000 live births in 2015 [4], considerable ongoing improvements in health worker skills and quality of care are required to reach the Sustainable Development Goal target of less than 25 per 1000 live births by 2030.

Trends in the global burden of disease from 1990 to 2015 show increased rates of chronic NCDs across LMICs both for children aged below and above 5 years [5], calling for interventions that more effectively identify and treat common chronic conditions, for example asthma which globally is the most common long-term health condition in childhood. In South Africa, the prevalence of asthma is 10% in 6–7 year olds and as high as 15% in 13–14 year olds, approximately half of affected children have severe uncontrolled symptoms and more than 30% have never been formally diagnosed [6]. Lack of chronic illness management training for nurses and limited access to doctors and equipment in primary health care facilities contribute to this situation, often leading to children with long term conditions bypassing these clinics and presenting at secondary level hospitals [7, 8].

The World Health Organizations’ (WHO) Integrated Management of Childhood Illness strategy (IMCI) [9], was developed to address the top causes of mortality in children under five, and is the standard of care in over 100 Low- and Middle- Income Countries (LMICs), including South Africa [10]. A 2010 multi-country review of IMCI [11] confirmed improvements in prescription accuracy, treatment and health service quality and a 2016 Cochrane review [12] found evidence of a reduction in neonatal and infant mortality. However, an evaluation of IMCI’s impact since its introduction in 1998 reported variable fidelity to the strategy’s guidance [13] limited training and ongoing supervision of primary care workers, (in South Africa usually professional nurses), and infrequent updating [14]. The IMCI strategy also does not address the health needs of children over 5 years or those with chronic conditions needing regular follow-up, and requires more complete integration of curative and preventive measures, including care for the well child. A key conclusion of WHO’s 2016 strategic review of IMCI stated that “with attention focused on specific child health areas such as immunization and communicable diseases, a holistic view of child health has arguably been lost inside the continuum of reproductive, maternal, newborn, child and adolescent health.” [12, 15]

In the Western Cape province of South Africa almost every public sector primary care facility employs an IMCI-trained nurse, and it is these nurses who attend to the majority of children’s healthcare care needs. At a series of meetings with key stakeholders in provincial paediatric health - primary care nurses, doctors, managers and educators, hospital-level paediatricians and policy makers - the growing gaps in knowledge and expertise for children at primary care level were recognised as well as a need to integrate well child routine care into the delivery of everyday paediatric primary care.

To help address these gaps, the Knowledge Translation Unit (KTU) in Cape Town, South Africa developed a paediatric version of its Practical Approach to Care Kit, (PACK) [16], intervention, comprising of a clinical decision support tool, training programme, and health system strengthening including enhanced supervision with regular updates as guidance and policies change [17–20]. PACK Child incorporates IMCI content but provides extended clinical guidance for the child older than 5 years (up to age 13), 16 long-term health conditions, an approach to the well child and additional non-life-threatening, yet common conditions.

The implementation and training elements of PACK Child are modelled on and complement PACK Adult [18], which was trialled and scaled up in South Africa to over 30,000 clinicians in more than 3,000 clinics [21–23], using a systematic, educational outreach training strategy and cascade model of implementation [19]. PACK is also being implemented in Botswana [24], Brazil [25], Nigeria [26] and Ethiopia [27], is available globally through a partnership with BMJ (pack.bmj.com) and is being localised for piloting in China [17].

Implementation of a more expanded programme like PACK Child alongside the long-established IMCI raised many legitimate concerns for policymakers, prompting a detailed process evaluation of the first pilot of the intervention in the Western Cape Province. These concerns were chiefly around whether, given the structural constraints, it was feasible to extend the scope of paediatric primary care delivery, and whether PACK Child would augment or undermine other priorities like IMCI, early childhood development, growth monitoring, preventive care and appropriate referral patterns.

In this article, we report on how the organisational and social context of paediatric primary care influenced implementation of PACK Child, presenting findings from an in-depth qualitative analysis of audio-recorded consultations to demonstrate the relationship between the delivery of PACK Child and the wider social context of paediatric primary care.

Previous research that has observed clinical consultations in LMICs has relied heavily on structured checklists to assess clinicians’ adherence to clinical protocols, and in paediatric consultations the focus has been on clinician adherence to IMCI guidelines [28, 29]. Whilst raising awareness of the extent of IMCI implementation, such research has isolated individual clinician performance from the contextual conditions that facilitate or constrain their behaviour, thereby offering limited insight into how to improve delivery of care. In the study reported here, we attempted to move beyond individualised explanations of clinician performance by tracing a relationship between the South African healthcare system, clinician-caregiver interactions and clinicians’ use of documentation, empirically exposing how the broader context of primary health care shaped the use of PACK Child in clinical consultations.

## Methods

The process evaluation used a linguistic ethnographic [30, 31] methodology, which combines strengths of linguistics and ethnography to systematically investigate human behaviour in context. Linguistic ethnography provides theoretical and methodological tools for analysing how the meaning of talk, text and objects shift over time and space. We have previously adapted this approach [32] to facilitate detailed investigation of complex healthcare interventions across macro, meso- and micro-contextual levels, drawing on Bronfenbrenner’s socio-ecological model of behaviour, which conceptualises individual action as a response to socially structured processes and characteristics, organised across a layered system of relationships [33].

Mixed methods were used including quantitative and qualitative data collection and analytic approaches. Qualitative data included observations of training sessions; semi-structured interviews with caregivers; clinician, policymaker and paediatric manager focus groups; documentation used in child consultations; and ethnographic observations of consultations and non-clinical areas in each facility. Quantitative methods included auditing of training attendance logs and clinician questionnaires completed 6 months after finishing the PACK Child training programme. In this paper, we provide a detailed report of findings from the qualitative analysis of our observations of non-clinical areas, observed and audio-recorded consultations, documents, and interviews and focus groups with primary healthcare (PHC) facility managers, senior paediatric managers and policymakers.

### Research setting

The setting for this pilot and process evaluation was 10 public- sector PHC facilities serving impoverished urban and rural communities in the Western Cape province, South Africa. Child health services within PHC facilities are provided for children aged 0–13 years. Phase One took place in a single facility, Phase Two in an additional three facilities and Phase Three in a further six facilities. The facilities were purposively selected to provide maximum variation of primary care delivery in partnership with the Western Cape Health Department’s People Development Centre, which oversees training and upskilling of public sector healthcare workers in the Western Cape – see Table 1. Factors considered important for observing variation included whether clinics were Ideal Clinic sites, (an initiative to improve quality of primary healthcare) [34], number of IMCI-trained nurses; differing levels of PACK Adult training coverage; and use of Integrated Clinical Stationery (an initiative to standardise documentation and facilitate continuity of care of children up to 6 years).

**Table 1:** Characteristics of PACK Child Pilot Facilities

Phase	Facility	Urban/Rural	Jurisdiction	No IMCI trained	No PACK Adult trained	Ideal Clinic Site	ICS pilot	Total Number of Staff	Number seeing children	Brief description of facility	Number completed PACK Child training	Average Facility Attendance (04/2016–04/2017)	
												Under 5	5–9 years
1	1	Urban	Municipal	11	26	No	No	26	4	1 triage area (ENA) 1 EN Immunizations 2 PN for sick child	15	1000	Not Available
2	2	Urban	Provincial	9	36	No	Yes	38	4	1 triage area (EN); 1 PN immunizations, 2 PN Sick child	9	1153	295
	3	Urban	Municipal	9	20	No	No	20	6	1 EN/PN Triage 1 EN Immunizations 1 EN PMTCT 2 PN Sick child	9	1000	Not available
	4	Rural	Provincial	5	15	Yes	Yes	16	8	1 EN Immunization/triage; 5 CNPs Sick child	6	700	Not available
3	5	Rural	Provincial	10	9	No	No	18	9	All staff see well child and sick child	13	1153	28
	6	Urban	Provincial	Not Available	Not Available	Yes	Yes	84	3	Dermatology and Asthma Clinic, Trauma, Recent well child visits (1 PN)	9	1944	199
	7	Urban	Provincial	1	40	No	No	64	2	1 PN Immunizations 1 PN Sick child	17	2061	13
	8	Urban	Provincial	3	20	Yes	Yes	20	2	1 EN Immunizations 1 CNP Sick Child	5	535	24
	9	Urban	Provincial	9	37	Yes	Yes	37	1	Currently mainly see children in trauma; but introducing well/sick childcare	8	144	227
	10	Urban	Municipal	7	12	No	No	12	6	1 EN Immunizations; 1 PN Sick children	8	977	18

CNP - Clinical Nurse Practitioner

EN - Enrolled Nurse

ENA - Enrolled Nursing Assistant

PN - Professional Nurse

PMTCT - Prevention of Mother to Child Transmission

### Data collection

To understand the **macro-contextual features** shaping delivery of the PACK Child intervention, interviews were conducted with managers at each PHC facility, and a stakeholder focus group with senior paediatric managers, policymakers and clinicians. Facility managers were asked about staff resource allocation to paediatric care, relevant policies, patient flow and perceptions of the PACK Child intervention for supporting the care of children. Senior paediatric managers and policymakers were asked about challenges of the current healthcare system and how they viewed the role of PACK Child in helping to address those challenges. We also conducted a documentary analysis of the structure and content of 1. The PACK Child guide, 2. The IMCI guide and checklist [9], 3. Integrated Clinical Stationery and 4. The Road to Health Booklet (old version) [35] to understand how the broader principles underpinning these different texts are operationalised to deliver paediatric primary care (see Additional files 1, 2, 3 and 4).

To understand the **meso-contextual features** shaping delivery of PACK Child, we drew on the PHC facility manager interviews, in conjunction with observations of waiting room and reception areas to understand the flow of patients through the facility. Using a qualitative observational framework, (see Additional file 5), the researcher recorded field notes of their observations of how children accessed care within facilities, from reception to different clinicians/providers.

To understand how clinicians’ use of PACK Child articulated with **micro-contextual features** of paediatric primary care we conducted observations and audio-recordings of clinical consultations with children and caregivers in each of the pilot facilities. Consultations were conducted in the language or languages the caregiver, child and clinician were most comfortable communicating in. Recordings of consultations conducted in Afrikaans and isiXhosa were translated and transcribed in English. A researcher (RC or JM) was present in the consultation room at the time of recording in order to observe and document how clinicians used PACK Child and other documentation during the consultation, as well as other relevant non-verbal behaviour which contributed to understanding the consultation.

### The PACK Child intervention

The PACK Child guide, which is aligned with recognised standards for guideline development [36, 37] is an evidence–informed, policy-aligned integrated clinical decision support tool, including algorithms that facilitate identification of likely diagnoses. The guide is designed to be adapted to LMICs globally, covering 63 common symptoms, including IMCI components such as diarrhoea and pneumonia, but importantly, it extends the scope of IMCI by focusing on children 0–13 years. It is also designed to address 16 long-term health conditions most commonly seen in primary care, as well as including a comprehensive approach to screening the well child. Routine care of the well child (see Additional file 1) includes measuring and interpreting growth, screening developmental milestones, checking immunisations, deworming, vitamin A, TB and HIV screening, as well as asking about the mental health of the child or problems in school. It also encompasses an assessment of the carer’s health including screening for psychosocial risk factors such as depression, violence in the home or financial difficulties. Routine care is intended to be sequenced after establishing the need for urgent care for the presenting symptom, but before definitive care for non-urgent symptoms. Clarity around prescribing scope is provided by colour-coding each medication according to prescriber level. Designed to promote the continuum of care required to break the acute episodic care cycle, the guide prompts routine care into every consultation. Its content reinforces the messaging of existing initiatives like the Road to Health Booklet Side-By-Side messaging [35], the First 1000 Days initiative [38] and the Nurturing Care framework [39].

Drawing on the successful PACK Adult training methodology [18], the PACK Child training programme used an onsite in-service cascade model (see Additional file 6) to be delivered in three phases for the pilot [19]. The first phase included one facility trained by a KTU trainer, the second phase included three facilities trained by two KTU trainers and the third phase conducted at six facilities was rolled out two by PACK Child Facility Trainers - government employees trained into the role by the KTU during a five-day off-site workshop. The training included eight onsite training sessions delivered weekly in the PHC facilities; this was expanded to nine during phase two of the pilot to include a “health systems session” focusing on patient flow and distribution of tasks among cadres in contact with children. The training was designed to target all cadres of clinicians at facilities, mainly nurses and doctors and emphasises the alignment of the PACK Child content to IMCI, integration of care for the child’s caregiver using PACK Adult, and to develop the skills of all clinical staff to encourage a multi-disciplinary approach to paediatric primary care.

During the course of the pilot, bi-weekly meetings were scheduled to feedback on the content of the guide and issues with implementation in practice. This provided a regular opportunity to capture further refinements and clarifications in the PACK Child guide and for the training development. One of the content developers attended the training sessions in the first phase to ensure the usability of the guide and identify challenges within the primary care setting.

### Eligibility and sampling

To be eligible for inclusion in the study, nurses and doctors needed to receive PACK Child training, and caregivers and children aged birth to 13 years needed to be receiving paediatric services at the selected facilities. Policymakers needed to be responsible for delivery of primary care in public sector PHC facilities.

Data collection for the process evaluation occurred concurrently with the three phases of the pilot, enabling analysis of Phase One data to inform the sampling strategy in Phases Two and Three. All facility managers were invited to be interviewed. On a typical day, 2–3 clinicians consulted children and all were invited to participate in consultation observations. Purposive sampling was planned in Phase One to select and recruit caregivers and children and was intended to be informed by diversity of child conditions, level of deprivation and the age of the child. However, consultation observations were dependent on which children presented at the facility on the day of data collection, and on nurses identifying and approaching eligible participants in the waiting room areas. In Phase One, nurses approached all eligible participants unless they decided it would not be appropriate to do so (e.g. child needed urgent attention and the mother was distressed). However, the limited number of children in Phase One who had a chronic condition or were older than 5 years informed identification and inclusion of these children in Phases Two and Three. To do so, we asked facilities to prioritise approaching caregivers of children who met these criteria. Similarly, the inclusion of only nurses in Phase One informed a proactive attempt to include doctors in Phases 2 and 3. We asked doctors in each facility if and when they consulted with children and then asked them to approach the caregiver and child about participation in the research. Senior paediatric managers, facility managers, nurses and doctors involved in the pilot; and policymakers from the City of Cape Town and Western Cape departments of health were invited to participate in stakeholder focus groups to review findings and facilitate discussions on the implications of PACK Child for wider implementation.

### Ethics

Ethics approval was obtained from University of Cape Town Human Research Ethics Committee, City of Cape Town Research Ethics Committee and the Western Cape Provincial Health Research Committee. Written consent for interviews and observations was obtained from all facility managers, clinicians and caregivers. Children over 7 years old were asked to give assent to their participation. Caregivers and children were asked to consent to interview and observation on the day they attended the clinic. Facility managers provided consent for observations of non-clinical areas. All participants were provided with written information about the research, informed that their participation was voluntary and that they could withdraw from participation at any time.

### Data analysis

To understand how PHC facilities were organised to provide child care, and the interaction between contextual features and intervention delivery, we firstly analysed manager and policymaker interview data, and field notes of our observations of waiting rooms and reception areas. All interviews were transcribed verbatim and thematically analysed. Themes and field notes from observations of waiting room areas were compared to identify and describe similarities and differences in the organisation and flow of patients across facilities. Secondly, we analysed the audio-recordings, transcriptions and researcher field notes of consultations to understand how macro- and meso-contextual features shaped, and were shaped by nurses' interactions with caregivers and children. A key focus was to identify instances of how use of PACK Child aligned with routine practice, providing “telling cases” [40] of the wider social forces structuring intervention delivery *at the point of delivery*.

Audio recordings of consultations were transcribed verbatim. A sub-sample was transcribed using conversation analytic conventions [41, 42] to provide detailed evidence of how clinicians' use of the PACK Child guide was negotiated within interactions with caregivers and children. We then inductively coded each transcript by activity, for example “eliciting the child’s presenting problem”, “physical examination”, or “advice giving”. We cross-referenced these against the field notes of the researcher’s observations to determine what documentation, if any, was used during each activity. This enabled us to obtain a broad picture of the structure of consultations within and between clinicians and facilities. We then coded clinicians’ questions according to their function as part of the clinical assessment process (e.g. asking about presenting complaint, wider information gathering) and the structural form of the question (e.g. polarised, content or alternative question). This enabled us to understand patterns of questioning within each activity and the role of PACK Child and other documentation in shaping clinicians’ questioning. Using data collected during Phase One, one researcher (RC) completed all the coding of activities and questions and a second (JM) independently coded a sample 10% of the data. A Kappa score was calculated in a first round of question coding (0.72–0.83). Disagreements in coding and coding categories were discussed, refined and then a second round of coding for a further 10% of questions conducted, revealing a high level of agreement (0.92–0.94). Finally we interrogated each transcript to understand the consequences of the consultation structure and question-response sequences for the ongoing interaction, how the clinician’s use of the PACK Child guide influenced the direction of the consultation, and how this use interacted with the use of other documentation.

### Data synthesis

The analysis of qualitative data was iterative, moving between data collection and analysis to test emerging theories, comparing how managers’ views related to actual implementation of primary care and use of PACK Child. For example, managers reported particular facility processes or protocols that we then compared with our observations of waiting room areas and clinical consultations. Instances of how PACK Child aligned with routine practice within consultations provided insight into the tensions between different contextual features which we could then investigate further in subsequent observations and triangulate with data obtained from manager interviews.

The synthesised data were then used to map macro-, meso- and micro-contextual features with a consideration of how national policy at a macro level impacted on the organisation and skill mix of staff at a meso level, and then ultimately how care was delivered to children at a micro level within consultations. By focusing on (mis) alignments to implementation and setting the PACK Child intervention within a contextual framework, we were able to make the transition from the identification of patterns of PACK Child use in specific facilities, to theoretical explanations of how different structural relations and mechanisms organise moments of delivery, facilitating generalisable inferences and predictions on how to optimize PACK Child for future implementation.

## Results

We conducted ten facility manager interviews (one per facility); one focus group with 24 stakeholders including clinicians, policymakers and senior paediatric managers (involving four smaller group discussions); ten observations (one per facility) of waiting room and reception areas; and 53 observations with audio-recordings of clinical consultations with children and caregivers, (Phase 1 = 16; Phase 2 = 13; Phase 3 = 24), totalling 18 h and generating 595 pages of transcripts. Forty consultations were conducted in English, eight in Afrikaans and five in isiXhosa. In Phase One, observations were interspersed between the eight PACK Child training sessions. Our analysis of these data identified clinicians reading aloud from the guide during consultations and difficulties using the guide alongside other medical documentation. This insight highlighted the importance of allowing time for clinicians to practise using the PACK Child guide and informed theoretical sampling of further observations in Phase Two and Three, which we timed to be conducted once the PACK Child training sessions had been completed at facilities. Following the high proportion of children presenting with acute infections in Phase One, we also attempted to sample children presenting with chronic conditions in Phases Two and Three. In Phase Three, one child with asthma and nine with eczema were included.

First we report macro-, meso- and micro-contextual features of paediatric primary care which had an impact on the integration of PACK Child at the point of delivery within consultations. In Tables 2 and 3 we have set out the macro and meso elements of context, with illustrative quotes from facility manager interviews. We then present extracts from the audio-recorded consultations, providing telling cases of how macro- and meso-contextual features were made salient by clinicians at a micro-contextual level, specifically in terms of how they used the PACK Child guide alongside other documentation and how they interacted with children and caregivers.

**Table 2:** Macro-contextual features of paediatric primary care in Western Cape, South Africa

Type of macro discourse, policy in play	Description
Integrated Management of Childhood Illness (IMCI) [9]	World Health Organisation’s IMCI is an integrated strategy that is targeted at reducing death, illness and disability, and promoting growth and development for children 0–5 years old. This strategy comprises both preventive and curative elements and has three components targeted at improving skills of primary care clinicians, health systems functioning, and family and community health practices. Principally delivered by nurses, IMCI is underpinned by a risk minimisation approach with the main aim of a provider-patient contact to ensure all children with danger signs are referred to the next level of care and provide reassurance that growth monitoring (and associated interventions e.g. Vitamin A) and immunisation take place. IMCI was introduced in South Africa in 1996 with a primary implementation focus on training and capacity building of clinicians [17]. In the Western Cape, the main manifestations of IMCI are the chart booklet, last updated in 2014, a training programme that targets professional nurses with the intention that they then see children, and the IMCI checklist (Additional file 2).
Primary Health Care Standard Treatment Guidelines (STG) and Essential Drug List (EDL) [43]	National level guidance comprising evidence based standardised recommendations for healthcare workers, in order to promote equitable access to safe, effective, and affordable health medications. These guidelines are not specific to children and include adults. There is limited guidance for neonates. Medication for children is recommended according to weight bands.
Expanded Programme on Immunization (EPI SA) [44]	Vaccination schedule updated in December 2015, implemented in provincial and municipal clinics, reducing in frequency after 18 months old up to 12 years. (https://www.westerncape.gov.za/assets/departments/health/vaccinators_manual_2016.pdf)
First 1000 Days Initiative [38]	The first 1000 days initiative aims to improve the nutrition of mothers and children during the first 1000-day window to ensure children get the best start to life and the opportunity to reach their full potential, starting from conception, moving through pregnancy, birth, and after the first 2 years of life (https://www.westerncape.gov.za/first-1000-days/).
Nurturing Care Framework [39]	The Nurturing Care Framework provides a roadmap for how early childhood development unfolds and how it can be improved by policies and interventions. It outlines: why efforts to improve health, well-being and human capital must begin in the earliest years, from pregnancy to age 3; the major threats to early childhood development; how nurturing care protects young children from the worst effects of adversity and promotes development – physical, emotional, social and cognitive; and what caregivers need in order to provide nurturing care for young children. (https://apps.who.int/iris/bitstream/handle/10665/272603/9789241514064-eng.pdf).
Nurse restrictions on prescribing	IMCI-trained nurses treating children are typically professional nurses with prescribing limited to treating acute symptoms only. Restrictions are in place for medications used to manage long-term conditions including inhaled corticosteroids for asthma and topical steroids for eczema. This results in referrals, with additional waiting time and contact, to clinical nurse practitioners or doctors for prescriptions to treat chronic conditions. *They need to treat their client according to their scope of practice. They can only prescribe according to a schedule, in fact according to the national EDL* [Essential Drugs List]*, where it says for this condition you can only give a certain treatment.* (Manager interview, Phase 1)
Chronic Illness Management and training for over 5 s	Nurses lack experience with chronic illness management at primary care level. “I: *How often do you come back for the asthma medication?* *CG: They didn’t put her on medication. They just said that I must see...look after. I must just keep an eye that her chest doesn’t tighten. I must bring her back immediately once this happens, or take her to the hospital, but they gave her an inhaler*.” (Caregiver interview, Phase 2) No specific guidelines or stationery for children above 5, until introduction of Integrated Clinical Stationery (Western Cape only – see below) “*Our clinical notes for the child older than 5 years we only use our clinical notes to make an entry we don’t have a form like this for children older than 5 years*.” (Manager interview, Phase 2)
Road to Health Booklet (RtHB) [35]	RtHB provided as patient medical record (Additional file 4), widely implemented in PHC facilities throughout South Africa. Underpinned by philosophy to support well child routine visits, continuity of information and provide a hand held record for caregivers that summarises the child’s health in the first 5 years of life. The RtHB was substantially revised and expanded to include health promotion messages in February 2018 (https://www.westerncape.gov.za/general-publication/new-road-health-booklet-side-side-road-health).
Integrated Clinical Stationery (ICS)	ICS was developed by the Western Cape Department of Health in 2015 following identification of a gap in clinical recordkeeping for children during a pilot audit in facilities. Facility records for routine care were found to be inadequate and IMCI checklists were scattered in patient’s folders in no particular order. ICS was designed to meet the need for facility and visit-based stationery that integrated well and sick child care. The stationery (Additional file 3) was piloted in five facilities from July 2016 and implemented in half of PACK Child pilot facilities at the time of this study. It has since been adopted for province-wide implementation.
Patient co-payments	In South Africa primary care is free at point-of-care including access to a wide range of medications and investigations for people of all ages. Hospital-level care is free for all pregnant women and children under 5.

**Table 3:** Meso-contextual features of paediatric primary care in Western Cape, South Africa

Institutional relations, workforce arrangements, local policy	Description
Services typically provided by municipal and provincial facilities.	Municipal PHC facilities typically provide well child services (i.e. growth monitoring, development screening and immunisations on appointment basis), and services for sick children aged 0–5 years. Provincial government facilities provide services to all sick and well children, with a high proportion of children aged 0–5 years.
Delineated clinical roles and multi-disciplinary working	Professional nurses trained in IMCI routinely see sick children under the age of five. In rural facilities, CNPs are typically the first clinician to consult a child. Doctors do not routinely see children other than those who are severely ill or attending follow-up clinics for TB or HIV care. Enrolled nurses typically run immunisation services and perform growth monitoring. I: *Do any doctors see children?* M: *Yes* I: *And is it only when they need they need extra assistance for cases, or do they see them regularly?* M: *Yeah, she prefers to see all those that are on ART and if it’s an emergency.* (Manager interview, Phase 3) Facilities frequently rotate their staff. M: “*Most are IMCI trained, on a regular basis I rotate but certain such as ARV and TB we cannot rotate as it is specialist. So that if someone is sick, others can float because of this. This ensures that the service is accessible, they all have the exposure*.” (Manager interview, Phase 1)
Caregiver seeking behaviour	Children with HIV, TB and other chronic conditions referred to larger PHC facilities (“community health centres”) M: *No, we don’t see many chronic we refer them to ((name)) Community Health Centre..* I: *So they don’t come here for repeat scripts or...* M: *No. So when they... it’s whereby maybe there will be diagnosed for the first time here, for instance if the client is coming, let’s say for eczema, that child will be treated for eczema. If the child maybe got severe eczema, then he willget transferred to((name of tertiary level hospital)) then ((name of tertiary level hospital)) will bring it back that this child needs to be treated like a chronic child. There that time will refer back because they’ve got all the resources at ((name of hospital)) unlike us.* (Manager interview, Phase 2)
Flow of children through facilities	**Registration:** For children requiring immunisations, care was typically accessed through an appointment system. Caregivers with a scheduled visit for an immunisation or growth monitoring arrived with their RtHB and placed it at a specific registration point with a box for appointments. Caregivers with children without appointments, coming for an acute condition or having missed scheduled visits, placed their RtHB in the non-appointment box at the registration desk. Patient records were subsequently retrieved by reception staff and placed in the weighing and triage area according to the order in which they arrived. **Weighing and triage area**: The weighing and triage area was either a room or open area where children were weighed and reason for the visit established. In the majority of facilities an enrolled nurse, with more limited clinical training than professional nurses, was allocated to the weighing area. Weights were measured but typically not plotted or used to interpret growth. Heights were not routinely measured in most facilities. Temperatures were taken if the child was feverish. Both sick and well children came through the weighing/triage area. Guided by the child’s RtHB, the nurse determined if the child required vitamin A and deworming medicine. Children were separated into emergencies, well, or sick child visits and allocated to the relevant nurse, typically based on the caregiver’s report of the presenting complaint, rather than through the nurse’s clinical assessment. In two facilities, this area also functioned as the immunisation room. In one facility, children were weighed and given immunisations in the consultation room. The triage area typically had a dehydration corner and breastfeeding area. **Well child:** Typically seen in the immunisation room. Caregivers and children waited in the waiting area to be called by the allocated nurse. The immunisations were mainly carried out by an enrolled nurse but in some cases, a professional nurse. Following the immunisation, the nurse plotted the child’s weight in the RtHB. Caregiver/child would then leave with their updated RtHB. **Sick child**: Between one and three nurses in each facility were allocated to see sick children. These nurses were generally professional nurses, who then reported to a clinical nurse practitioner or doctor. In two facilities, sick children were prioritised and seen before adults. If the child was classified as an emergency, they went straight to the trauma room. Most of the consultation rooms for sick children had a stock of medication to dispense but, in some cases, caregivers had to go to the pharmacy to collect their prescription. In one facility, caregivers/children were required to see approximately four people if also needing treatment for Prevention of Mother to Child Transmission (PMCT) of HIV, including nurses to: triage, give immunisations, treat acute conditions and deliver PMTCT.
Local protocols/documentation for treating children	- Immunisation, developmental screening, deworming, vitamin A supplementation, health promotion and growth monitoring: RtHB and IMCI checklist or Integrated Clinical Stationery (ICS) - Sick child (0–5 years): IMCI checklist or ICS - Sick child (6 years and above): ICS. - Referral forms Provincial departments of health require facilities to complete stationery with IMCI components for consultations with children 0–5 years. ICS stationery also includes information about family, social context and chronic conditions (other than HIV and TB). ICS pages designed in columns to track previous visits. Province applies IMCI audit tools to determine clinician alignment with IMCI guide and whether facilities are treating expected numbers of children. IMCI audit data fed back to national Department of Health and WHO figures on child mortality.
Pattern of care-seeking from PHC services	The primary health care service offering is chiefly structured as preventive care (immunization and growth monitoring) and curative (acute illness), both in children under 5, which over time has shaped care-seeking patterns at community level. Children with chronic illnesses such as asthma rarely receive routine care in primary care, and are often referred to secondary and tertiary services which are usually some distance from communities, or the Community Health Care centres where there is little continuity of care outside HIV and TB treatment programmes. This perpetuates poor care seeking outside acute episodic illnesses and does not grow an understanding of regular, planned care for children with long-term health conditions. Caregivers frequently make use of an extensive network of private general practitioners who provide acute episodic care and medication for a fixed fee, but rarely chronic care. I: *Do you think many children come with a chronic illness problem, or do they come with an acute symptom?* M: *The majority is acute symptoms, but here and there we have babies that is on asthmatic treatment also, but the majority is acute, and the majority is pneumonia cases, severe pneumonia cases.* (Manager interview, Phase 3)
Referrals and continuity of information	Facilities reported rarely receiving feedback from hospitals following patient referrals. Caregivers receive discharge summaries from referral centres but do not routinely bring them to PHC facilities.

**Fig. 1:**
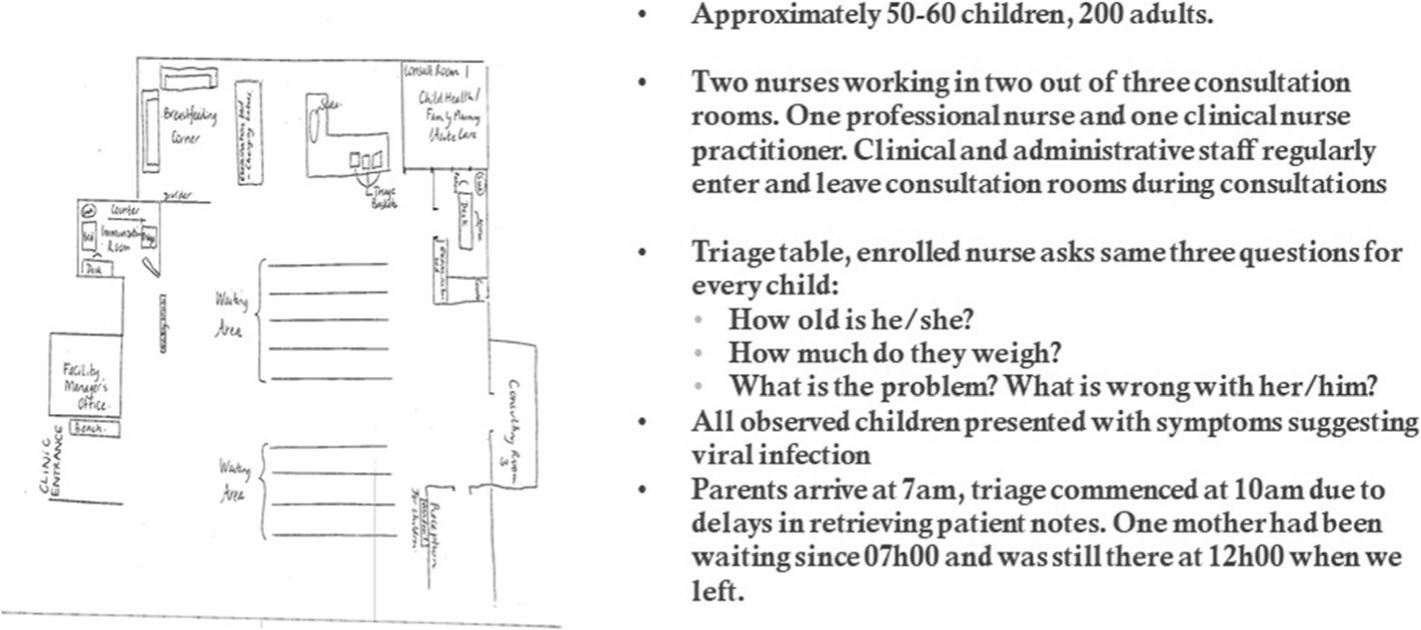
Observation of waiting room, triage and reception area

A particular challenge was how clinicians worked to incorporate the training and guide alongside pre-existing practice, while complying with provincial requirements to complete IMCI checklists and in half of the facilities, new Integrated Care Stationery for auditing the clinical management of children aged under five. Figure 1 is an extract of observational field notes recorded by a researcher over a three-hour period observing a facility waiting room area during Phase One. The diagram shows lines of benches, three consulting rooms, a triage area staffed by enrolled nurses and a breastfeeding corner. The field notes report a two-hour period of observing the triage desk. Triage commenced 3 h after caregivers and children arrived at the facility, following delays in retrieving the child’s medical notes. Children presented as well or with acute symptoms, typically a rash, sore throat or fever. Children were weighed at the triage desk. The enrolled nurse did not plot the weight or interpret the growth of the child. Once caregivers had answered the same three questions (i.e. age, weight, problem) there was no further clinical assessment until their consultation with a nurse. These field notes represent a broader pattern we observed, of caregivers attending facilities with children aged 0–5 years when they had acute symptoms, or needed immunisations and their growth monitoring; and PHC facilities predominantly oriented to deploying nursing staff to consult and treat children’s symptoms as discrete episodes with little consideration of the child’s long-term health needs.

### The impact of the organisational context on the use of PACK Child during consultations

Clinicians participating in consultation observations included clinical nurse practitioners (*n* = 17), professional nurses (*n* = 11), doctors (*n* = 3) and enrolled nurses (*n* = 2). Three children were aged under 2 months, 37 between 2 months and 5 years, and 13 children were 5 years or older. Reasons for seeking a consultation for their child predominantly included acute symptoms suggestive of a viral infection, including rash (*n* = 14), cough (*n* = 7) and other respiratory symptoms (*n* = 7). Ten children presented with likely long term conditions – eczema (*n* = 8) and asthma (*n* = 2). Remaining reasons included eye symptoms, gastro-intestinal problems, injury and visits for immunisations. We now examine how the macro- and meso-contextual features impacted on clinician-caregiver-child interactions. In doing so, we are observing an interaction at a micro-contextual level, between the approach of PACK Child with a focus on children aged 0–13 years covering acute and long-term health conditions and screening of the well child, and the existing healthcare system where IMCI policy [9] and use of the RtHB [35] are embedded, and ICS is being introduced.

#### Clinical assessment questions

In our sample of 53 audio-recorded consultations we identified and coded 1218 clinical assessment questions. Table 4 displays four important features about the nature of these questions in our sample. Firstly, the three highest number of question types were oriented to topics required by IMCI – acute symptom management (wider information gathering and reported complaint) and growth monitoring, immunisations and questions about feeding, making up 56% of all questions. This partly reflects the characteristics of our sample with 37 out of 53 children presenting with acute physical symptoms but also reveals the orientation to IMCI policy within consultations. Secondly, 84% of psychosocial questions were delivered as polar questions, with only 14% delivered as content questions (i.e. questions with “what”, “where”, “why”, “how” formulations). Polar questions [45] are questions that are either interrogative or declarative and are designed to prefer either a “yes” or “no” response. In the process of clinical assessment, clinicians’ use of polar questions have also been shown to frequently prefer no problem answers [33, 46]. For example, “And she is weeing ok?” is a declarative question designed to prefer a yes and rule out dehydration, whilst the inclusion of “at all” tilts the interrogative “Has she vomited at all?” to prefer a no and the absence of vomiting. Applying this to questions designed to elicit potentially sensitive psychosocial issues, the high proportion of polar questions relative to content questions suggests that clinicians did not design questions which invited disclosure of psychosocial problems around the child. Thirdly, the number of questions about long-term health conditions (other than TB and HIV), located in 18 out of the 53 consultations shows that clinicians sometimes identified symptoms as markers of potential chronic conditions, prompted by the routine care and long-term condition pages within the PACK Child guide. Questions included those aimed at determining if the child had an allergy, asthma, mental health or behavioural difficulties. Finally, we identified only six questions that elicited caregivers’ concerns, ideas or expectations and only nine questions that assessed past medical care (excluding TB and HIV). While the PACK Child intervention does not specifically prompt clinicians to elicit caregiver’s perspectives, this finding suggests that the clinicians in our sample did not habitually ask questions that attempted to gain a picture of the child beyond the specific problem presented on the day.

**Table 4:** Clinician question coding by type and structural form

			Structural form of Question			
Question Type	Question Example	Number of consultations ***N (%)***	Polar ***N (%)***	Content ***N (%)***	Alternative ***N (%)***	Total ***N***
Wider information gathering	*“Any symptoms that you are having concerns about, besides his skin now?”*	41 (77)	194 (77)	50 (20)	7 (3)	251
Assessing feeding/growth monitoring/ immunisations	*“So you are no longer breastfeeding?”*	37 (70)	165 (69)	69 (29)	6 (3)	240
Asking about reported complaint	*“Coughing for how many days?”*	45 (85)	119 (61)	67 (34)	9 (5)	195
Eliciting psychosocial issues	*“And you do you have support from the child’s father?”*	29 (55)	143 (84)	24 (14)	4 (2)	171
Asking about HIV or TB	*“Have you tested for HIV when you were pregnant?”*	36 (68)	95 (71)	32 (24)	7 (5)	134
Asking about treatments	*“What tablet did you give?”*	31 (58)	86 (72)	27 (23)	7 (6)	120
Asking about other long term health conditions	*“Is he a known asthmatic?”*	18 (34)	50 (89)	6 (11)	0 (0)	56
Asking about family planning	*“And you yourself are you on any family planning mommy?”*	19 (36)	26 (72)	9 (25)	1 (3)	36
Assessing past medical care other than TB/HIV	*“So the child hasn’t been treated at any other institution before for anything, for this problem?”*	7 (13)	6 (67)	2 (22)	1 (11)	9
Eliciting caregiver concerns, ideas, expectations	*“Is there anything that you would like to ask?”*	5 (9)	6 (100)	0 (0)	0 (0)	6
Total			890	286	42	1218

Notes: This table shows the number and proportion of consultations for each question type in the sample of observed consultations. It also shows the number and proportion of different structures within each question type. Polar questions prefer a yes or no response. Content questions (or Wh- questions) are open questions inviting new information whereas alternative questions present two or more options embedded in the question. Proportion of consultations is a percentage of all 53 consultations. Proportion of polar, content and alternative questions are percentages within each question type category

Taken together, these different features of clinical assessment questions suggest that clinicians were negotiating an institutionalised practice to treat symptoms as acute episodes that need to be assessed according to level of risk on the day, with a different approach which views symptoms as potential indicators of underlying conditions. In doing so, clinicians could be seen to be operating in a transitional space between a risk minimisation approach on the day to risk minimisation over time. The challenge in making this transition is most clearly seen in the use of polar questions to elicit psychosocial issues. Rather than viewing the predominance of polar questions designed to limit disclosure of psychosocial issues as a failure of nurse performance, we can see these questions as a manifestation of the wider healthcare system in which they were operating. Working within an everyday context where large numbers of children from impoverished backgrounds with high rates of adversity present with acute symptoms that clinicians need to assess for risk, monitor growth, check immunisations and feeding in busy, time-constrained consultations with limited confidential spaces and referral resources, it is unsurprising that nurses adopted to phrase these questions in such a way that it limited the possibility of disclosure of sensitive psychosocial problems.

### Introducing the PACK Child guide into routine consultations

An issue for the delivery of PACK Child consultations is how clinicians negotiated different routine care and symptom-based activities, various sections of the PACK Child guide, whilst also completing necessary documentation. The extract in Table 5 provides a “telling case” of this negotiation [40], taken from a consultation conducted in one of the facilities participating in Phase Three of the study, which involved a nurse using PACK Child to manage and treat a three-year-old child presenting with a cough.

**Table 5:** Nurse navigating PACK Child with IMCI checklist and RtHB

Nurse (N) or Caregiver (CG)	Nurse/caregiver talk :: Elongated vowel [] Overlapping talk (1) Timed pause, (.) less than 1 s. ^°°^ Hearably quieter speech CAPITALS denotes hearably louder speech Underlined talk indicates spoken with emphasis Heh heh denotes laughter (()) Further information	Use of PACK Child guide, IMCI checklist and RtHB
**N**	O::kay a::nd uh (.) feeling hot at night? Or during the day?	N writing on IMCI checklist under “Fever” Yes or No
	(1.0)	
**CG**	[No::]	
**N**	[No] okay and u::m (.) can I see your hand and the babies hand? ^°^I am going to try to be quick^°^	N checking ‘Anaemia’ on IMCI checklist
	(??)	
**N**	Okay thank you. An::y (.) what is your HIV status Si:si::? ((Sister in isiXhosa))	N working through IMCI checklist “Consider HIV infection”
**CG**	[Negative]	
**N**	[Your HIV]? Negative	
**CG**	Huh	
**N**	When, when did you, whe:n did you?	
**CG**	You are the one who did la:st month.	
**N**	Heh heh heh [heh heh heh] ((Nurse realises she forgot that CG has already taken HIV test))	
**CG**	[Heh heh heh] When I come with ((name of child))	
**N**	Okay. O::kay. U:H How old is this baby FIRST?	
**CG**	She is 2 years three mo:nths	N opens PACK Child to content page
	(3.0)	N looks at RtHB
**N**	O::kay, we go to a content page which is u::h page um (2) u::hm 50 for cough and also we go for routine care which is page u:h 14. She is, how old is she now?	N opens PACK Child routine care page to check what she needed to do.
**CG**	Two:: yea::rs	
**N**	Mmm	
**CG**	A:nd 3 months	
**N**	Two years and thre:e months. Two years is here, we must check the weight. Let’s see the weight, the weight is 16 and where is he:r card? Is here ((child coughs)). HAIBO ((surprised expression in isiXhosa)) SISI you are coughing ne: ((Afrikaans particle word meaning “isn’t that so” used for emphasis))	N reading from routine care page N searching for RtHB
**CG**	Mm	
**N**	16 point (.) plot the wei::ght. 16 point 6. She is 2 years a::nd?	N plotting weight in RtHB
**CG**	Three months.	
**N**	And three mo::nths (1) March April May June Ju:ly (2) and is 16 point six (2) hmm (12.0) sixteen (.) which is 16 point 6 (.) Yoh! She is growing very well ne	N showing CG that child is growing well.

Consultation from a Phase 3 PHC facility with a mother and three-year-old girl presenting with a cough she has had for 3 days. The nurse begins the consultation using the IMCI checklist where she documents the cough as the presenting symptom, enquires about the presence of diarrhoea and the caregiver shows the nurse the child’s skin rash. The extract begins after 2 min into the consultation

The transcript of this consultation shows the predominance of different medical documents and guidelines which clinicians had to navigate within the consultations, in this case the IMCI checklist, RtHB and PACK Child guide. Following a question about the duration of the child’s cough, the extract begins with the nurse using the IMCI checklist to complete three tasks, asking about the child’s temperature, examining the child’s hands and checking the mother’s HIV status. For each of these tasks we can see how the IMCI checklist plays a key role in steering the nurse questioning and sequence of activities within the consultation. At 2 min and 47 s, and after completing the IMCI checklist, the nurse opens the PACK Child guide for the first time whilst also referring to the RtHB. The nurse identifies which page in the guide deals with coughs but also the routine care page, where each PACK Child consultation is intended to begin. The nurse selects the routine care page and checks what needs to be covered in the consultation. Prompted to check the child’s weight the nurse then searches for the RtHB and plots the child’s weight as required.

Following the end of this extract the nurse then continues to check items prompted on the PACK Child routine care page, including TB risk, immunisation status, vitamin A and deworming. After completing these tasks at 10 min and 30 s, the nurse states that “we are going to the real problem now” and turns to page 50 in the guide to address the child’s cough. The numerous pauses in this extract, elongated vowels by the nurse and the sound of the nurse searching for different pages (as heard on the recording), indicate the work the nurse is doing to navigate and complete all three documents and demonstrate the central role of documentation within paediatric consultations.

While this extract clearly shows the burden of documentation within paediatric consultations, it also reveals a broader tension between IMCI policy [9], oriented to acute episodic care, and PACK Child which is attempting to embed routine care into every consultation, with a view to longer term care over time. As we have argued, these broader policy and institutionalised tensions play out a micro-contextual level within clinician-caregiver interactions, offering explanations that go beyond a focus solely on individual clinician’s competency.

#### Responding to and managing long-term conditions

In assessing clinicians’ ability to use PACK Child to facilitate diagnosis and management of long term health conditions, an important task was how clinicians responded when conditions or psychosocial problems were identified. Tables 6 and 7 contain extracts from two consultations conducted in Phase 3 facilities; telling cases which provide insight into how macro- and meso- contextual features constrained or enabled clinicians to respond to the needs of children.

**Table 6:** Negotiating caregiver report of behavioural and family problems

Nurse (N) or Caregiver (CG)	Nurse/caregiver talk ↑ High pitch Underline – spoken with emphasis […] sequence of consultation not included	Use of PACK Child guide
**N:**	Is is his own mother still involved in his life?	Opens to contents page
	(0.7)	
**CG:**	↑Noo::	
**N:**	[She doesn’t …]	
**CG:**	[She’s her father] is her father is raising two kids of hers those two are working now. (1) Her father is also a FAS ((Fetal Alcohol Syndrome)) baby (1) I say every father gets his packet.	
**N:**	Mhm	
**CG:**	They gave him she had tw::o, three children minimum, by a gu:y, two boys and a a girl and she dropped the children by the father and she left (1) she’s now she is a year gone from there now.	
**N:**	Mhm	
**CG:**	And here he is if she comes she just come and then he fights with her (1.5) because she pu::lls him and they’ve got that anger. And and I tell her she mustn’t pull him because he don’t like people to pull him around, and she got a habit of that ‘Kom met my saam’, ‘come with me now’, you know? (1.5) so many times and I told him, ‘you mustn’t fight with a mother’ that is still your mom (1) irrespective.	
	(1.5)	
**N:**	So you said he is got sore throat?	

In a Phase 3 facility a 12-year-old boy presents for an appointment with an ear problem. During the consultation the caregiver voluntarily discloses that the child has a history of Fetal Alcohol syndrome, takes Ritalin for behavioural problems (implying likely involvement of tertiary service because of limited access to Ritalin), and has a difficult relationship with a largely absent mother. Despite evidence that the nurse is listening to the caregiver’s concerns about family life, the nurse does not discuss the child’s use of tertiary or social services and she does not refer to the PACK Child guide which includes pages on how to manage behaviour and anger problems as well as potential child abuse

**Table 7:** Using PACK Child to make a transition from acute symptom to chronic illness management.

In a Phase 3 facility a four-year-old girl reports to the clinic with a cough, recurrent wheeze and at the beginning of the consultation the mother reports that the child has asthma. The child was nebulised before the consultation, and no wheeze is heard on auscultation by the nurse. The expected route through the PACK Child guide would be to start with the routine care page for every visit, then refer to the wheeze symptoms page to manage acute symptoms, finishing with the asthma routine care in the long-term health condition section. The clinical nurse practitioner initially refers to the cough page in the PACK Child guide and then navigates to the recurrent wheeze page. She diagnoses the child with allergic rhinitis and prescribes a nasal spray and cetirizine. The mother reports having enough “pumps” but the nurse doesn’t clarify what this includes and prescribes budesonide metered dose inhaler, advising the caregiver that it needs to be taken twice a day and Ventolin (salbutamol) used when necessary. The nurse only briefly refers to the asthma routine care page and does not ask the caregiver about the child’s history of exacerbations or hospitalisations. However, following use of PACK Child the nurse advises the caregiver to book a review appointment in 3 months.		
	**Nurse/caregiver talk** (…) unclear talk	**Use of PACK Child guide**
**CG**	She is asthmatic, she comes here for oxygen. I do put her on the nebulizer at home, but it doesn’t actually help, because she was coughing all week. I had her on the nebulizer last night, but then this morning I told her it would be better if I bring her for the oxygen. They did examine her, they gave her a dosage. So they gave her one this morning. Like the cough just didn’t want to go away	
	(…)	
**N**	Okay, the mom is complaining of a cough, so I go to the contents page.	
**CG**	(…) She’s forever chesty (...).	
**N**	The child with breathing problems may have noisy breathing, wheeze. Did she have a wheeze this morning, before they nebulized her?	Checking PACK Child cough page
**CG**	Last night they nebulized her.	
**N**	And this morning I saw that they gave her a nebulizer?	
**CG**	Umm no, no::t this morning. Probably they gave her oxygen, yes.	
**N**	But it’s a nebulizer.	
**CG**	Okay	

The extract in Table 6 demonstrates a lack of information provided by tertiary or social services surrounding the child’s behaviour and problems with his parents, with the nurse needing to decide how to respond within the constraints of a time-limited consultation which also required her to tackle the child’s sore throat symptoms. Despite the availability of pages within PACK Child that guide the clinician on how to manage symptoms of behaviour, anger and abuse, thereby offering the opportunity for the nurse to support continuity of care between primary and tertiary services, the nurse instead redirects the focus from a complex set of psychosocial issues back to the acute physical symptom.

In contrast, the extract in Table 7 illustrates a nurse operating in the transitional space between a health care system structured to focus on treating acute symptoms and PACK Child that supports ongoing care of long-term conditions. The clinical nurse practitioner, using the PACK Child guide is able to prescribe an inhaled corticosteroid for asthma, successfully diagnose comorbid allergic rhinitis, and books a follow-up appointment for the child. However, the nurse doesn’t explore which inhalers the child is already using, follow the guide as instructed in the training programme, or ask questions about previous exacerbations or hospitalisations.

Tables 5, 6, 7 provide “telling cases” [40] which empirically expose a broader tension between a primary care system oriented to acute symptom management and PACK Child’s focus on care for the child over time, illustrated through nurses’ use of different documentation (Table 5); tensions between PACK Child’s orientation to routine care and psychosocial issues, and a healthcare system oriented to acute physical symptoms (Table 6); and nurses having some success in using PACK Child to treat chronic conditions but struggling to orientate to a view of the child’s condition over time (Table 7). These instances triangulate with the ethnographic observational data (Fig. 1) that showed a predominance of children under 5 presenting at facilities with acute symptoms; interviews with facility managers who reported children with chronic illnesses were routinely referred to tertiary level hospitals (Table 3); and the analysis of questions (Table 4) that found clinicians predominantly asking questions required by IMCI, psychosocial questions designed to minimise rather than invite disclosure of problems, and a scarcity of questions that attempted to elicit caregiver perspectives or the child’s medical history.

## Discussion

The PACK Child intervention was developed to address the limitations of IMCI in tackling preventable and treatable conditions in children, expanding a focus from under-fives to children aged up to 13 years and those living with long term health conditions. However, implementation of PACK Child needs to take place within a primary healthcare system that primarily deploys professional nurses focusing on conditions covered by IMCI, and restricts nurse prescribing for common long-term health conditions like asthma and eczema. This presents a number of challenges for how best to embed an intervention into routine practice that aims to provide more holistic care across age groups, a spectrum of acute and chronic conditions and constellations of clinical and psychosocial needs. The mapping of macro- and meso-contextual features, observation of patient flow within waiting room areas, the profile of patients within our sample and the analysis of consultations provided insight into how these challenges are rooted in primary care facilities that are institutionalised to receive and treat children 0–5 years, predominantly for acute symptoms, to monitor growth and ensure immunisations are up to date.

The extracts from clinical consultations presented within this article offer insight into how clinicians struggled to integrate the use of PACK Child alongside either the IMCI checklist, ICS and RtHB, producing disjointed consultation structures. This finding highlights that caution should be exercised when asking clinicians to manage different documentation within consultations. However, to focus solely on the difficulties of managing documentation within consultations would be to reduce the interpretation of findings to individual clinician performance, thereby isolating the clinician’s behaviour from the wider healthcare system in which that performance is structured and brought into action. Such a reduction in focus has typically been a limitation of research that has assessed nurse adherence to IMCI [28, 29, 47, 48]. A more important conclusion to be taken from the extracts we have reported here is that they reveal tensions between broader policies that are invoked by clinicians when using these different documentation in their interactions with caregivers and children. Firstly, the IMCI checklist was designed to operationalise a risk minimisation policy aimed at tackling the leading causes of child mortality. The clinician must record information primarily using tick boxes that inevitably drive the design and sequencing of clinical assessment questions to rule out the presence of life-threatening conditions. Secondly the ICS, which incorporates IMCI risk minimisation components, represents an extension of IMCI to provide continuity of documentation, using columns to track previous visits. It was also designed to complement PACK Child, with space to record a range of long-term conditions, to support ongoing routine care of children 0–5 years (a separate form for children aged over 6 years), as well as addressing the psychosocial context and risks surrounding the child. The PACK Child guide and ICS are therefore documents that embody a broader agenda to tackle a perceived absence in the continuity of information for children, an assessment of progress of the child over time and the importance of tracking long-term health conditions as the child develops. A different approach to the consultation is therefore required, utilising questions that orientate more closely to facilitating diagnosis of underlying conditions, track the child’s medical history and enable disclosure of potentially sensitive psychosocial issues. Finally, the RtHB is also designed on a principle of ensuring continuity of information including growth monitoring charts, largely duplicating information within the IMCI and ICS documents to be kept by the caregiver.

The clinician, when using these different documents in one consultation is therefore navigating his/her way through these different policies recontextualised at a micro-level into different consultation structures and question formats which may not be neatly aligned. The interactions we observed are therefore manifestations of these misalignments, including clinicians using polar-declarative questions to elicit psychosocial issues, avoiding difficult social problems in favour of acute physical symptoms, and interactions that display clinicians attempting to make a transition from a focus on symptoms as discrete episodes to underlying conditions that need to tracked and managed over time. The point being made here is that whilst streamlining documentation is important for enhancing the potential for comprehensive care, it needs to be supported by a healthcare system that is structured to minimise risk and support wellness of children and families over time alongside a risk minimisation policy for acute illness episodes.

### Optimising the implementation of PACK Child

By investigating the use of PACK Child within a broader contextual framework we were able to develop hypothetical propositions for optimising the implementation of PACK Child on a wider-scale. Importantly, and in contrast to previous observational research of paediatric primary care in LMICs [28, 29, 47, 48], this approach facilitates the generation of strategies for strengthening the healthcare system that may greatly enhance the impact of training and the practice of clinicians within paediatric consultations.

At a macro level, our evidence strongly suggests that the current paediatric care offering urgently needs revising to facilitate enhanced skills, knowledge and deployment of nursing staff with the right levels of expertise to better address the acute illnesses of children of all ages but also to more adequately treat and support children living with long term health conditions. Such conditions may include a complex mixture of physical, behavioural, psychological and social problems that are being sustained and perpetuated over time. PACK Child was designed to meet these needs if structural changes facilitate a clinical practice that orientates to continuous rather than episodic care. Previous evidence has already emphasised the need for a more systematic implementation programme of IMCI [49, 50], and for not relying solely on training to improve quality of care. Our evidence supports this recommendation but emphasises that without reorienting primary health care towards a view of the child and family evolving over time, the full range of health and social needs of children will remain unaddressed [13].

At a meso level, the capacity for clinicians working in a busy facility environment to deliver care that adequately addresses a complex array of needs, whilst also meeting provincial requirements to complete documentation is clearly challenging. In addition, while in theory comprehensive services are available for selected conditions at facilities, caregivers and children often have to see multiple clinicians in order to receive the care they require. Additional touchpoints are likely to entail increased loss to follow-up, are not person-centric, may be an inefficient use of clinical resources as well as presenting infection control risks for children. The PACK Child guide is designed to support clinicians to provide more comprehensive care without unnecessary duplication but requires facilities to consider how best to deploy staff resources to meet this objective. The inclusion of a “health systems strengthening” session within the PACK Child training programme (Additional file 6), which asked clinicians to examine the distribution of roles at different points in the facility visit, represents an initial attempt to streamline care. The evolving use of digital technologies also offers potential for supporting streamlined care and ensuring continuity of information across contacts, and lessons learnt from piloting digitised versions of PACK guidelines have already been reported [20].

Whilst the PACK Child intervention was designed to incorporate routine care into every consultation our findings highlight the need to carefully consider how to deploy resources to effectively meet the range of children’s needs and to prioritise requirements of routine care to make this activity more efficient. Our findings illustrate that such challenges are particularly pertinent for screening and responding to psychosocial issues surrounding the child, demonstrating that asking caregivers about psychosocial issues may have limited impact when embedded as part of a list of routine screening questions. Similarly, clinicians need to know how to respond appropriately when psychoscocial issues are disclosed. As well as clearly mapping social and community resources before introducing PACK Child at a facility, alternative solutions to routine screening within consultations could lie in mobilising community health workers to build relationships with families and ask more specific and targeted questions that might support the child more effectively over the long term [51].

At a micro level, detailed consideration is required regarding how to better integrate medical record stationery alongside PACK Child, so as to streamline and free up consultation time, which will allow for more involvement of caregivers and children. Consultations have to be optimised to maximally benefit the child, not just in terms of their specific problem on the day but an approach that enables the child’s history and onward referrals to be tracked and followed on through at subsequent consultations and with different professionals. In this respect the ICS offers advances over the IMCI Checklist and has been adopted for province-wide implementation since completion of this study. Caregivers provided detailed accounts of their children’s healthcare utilisation and symptoms in this study, and should not be overlooked in systems that cannot guarantee continuity of provider.

### Strengths and limitations

This process evaluation was to our knowledge the first study in LMICs to use a linguistic ethnographic methodology to map salient macro-, meso- and micro-contextual features of child health systems and attempt to identify relationships between different contextual features and the implementation of a complex healthcare intervention within clinical consultations. By analysing clinician-caregiver-documentation interactions and working laterally across different data types, we were able to generate theoretical generalisations regarding the relationship between the broader context of South African healthcare and the specific moments of delivery in which PACK Child was being introduced. A particular strength of this analysis was the extensive use of observational data and identification of misalignments to delivery, functioning as telling cases that expose broader tensions between the existing healthcare system and the PACK Child intervention. This presented a significant advantage over solely relying on stakeholder perspectives of delivery in order to understand the realities of embedding a new complex healthcare intervention into existing practice.

Our observations of consultations were likely affected by the researcher’s presence and limited by the timing of data collection, which was both during and immediately following completion of the PACK Child training programme. This meant that we were observing clinicians who had limited time to develop their skills using all components of the PACK Child guide and may have been anxious about the researcher judging their performance. However, our focus was not solely on the extent to which clinicians followed each element of the guide, but more specifically *how* their use of the guide and interaction with caregivers and children was a result of the contextual conditions under which they were working. As we have described this included a negotiation of PACK Child alongside other documentation.

We faced some difficulties recruiting and selecting a diverse group of children and caregivers as we were reliant on which children presented on any given day and on the availability of nurses to enable us to observe consultations. Only ten of the 53 consultations were for children presenting with chronic conditions and only two of these were scheduled visits. Two PHC facilities held dedicated asthma and eczema clinics and it is possible that other scheduled visits produced different behaviours to the ones we observed. However, the breadth of observational, documentary and reported evidence we obtained, which demonstrates an institutionalised orientation to acute symptom management on the day of presentation, suggests that our study was not lacking in evidence of a practice where chronic illnesses were systematically identified and managed at a primary care level.

This research was carried out in the Western Cape province, inevitably limiting the transferability of the findings to parts of South Africa with fewer doctors and clinical nurse practitioners, or indeed to other LMICs with differing healthcare systems. However, a key objective of this study was to identify how best to optimise the delivery of PACK Child, generating recommendations for both the design of the intervention and the organisation of paediatric care more generally. For example, while Integrated Clinical Stationery is a Western Cape initiative, our findings emphasise the need for caution about the form and quantity of documentation generally, which may function to perpetuate risk minimisation and reduce person centredness, applicable no matter what stationery is used. The depth of the analysis within this study unpicked relationships between intervention and context that are far-reaching beyond the specific documentation, skills and resources that we observed in the Western Cape, offering wider theoretical generalisability, both in South Africa and low and middle income countries generally.

## Conclusions

More than two decades since IMCI was introduced, our findings reveal that a review of the priorities for paediatric healthcare are now required, alongside a detailed consideration of how different policies are translated into practice at an institutional level. Health systems need to buy into a transitional space where both risk minimisation and longer term care for the child over time can be more readily accommodated through review of who provides what care in what consultation. This includes making a shift from risk minimisation on the day to risk minimisation and promotion of wellness over time. Once such an approach is in place facilities will arguably be better placed to tackle a range of problems including complex psychosocial issues that may surround the child. The PACK Child guide and training programme could be instrumental in initiating such a shift on the ground within the realities of everyday primary care. To maximise its potential requires a healthcare system that makes a similar shift from acute illness paradigm to a larger remit of enabling the child to survive, thrive and transform.

## Supplementary information

**Additional file 1.** Child > 2 months old: Routine Care. Sample of routine care page from PACK Child guide.

**Additional file 2.** Sick Child Age 2 months to 5 years. Sample of IMCI Checklist.

**Additional file 3.** Integrated Stationery for children < 5 years. Sample of Integrated Clinical Stationery used for children less than 5.

**Additional file 4.** Road to Health Booklet. A sample of the road to health booklet (version used during the pilot).

**Additional file 5.** Observation guide-Non Clinical Areas. Observation guide for non-clinical areas.

**Additional file 6.** PACK Child Training Programme and Cascade Model. Model of Training and Cascade Model.

## Data Availability

The datasets generated and/or analysed during the current study are not publicly available due to data transcripts including personal participant information not suitable for sharing, but are available from the corresponding author on reasonable request.

## Abbreviations

HIV: Human Immuno-deficiency Virus
ICS: Integrated Clinical Stationery
IMCI: Integrated Management of Childhood Illness
KTU: Knowledge Translation Unit
LMIC: Low and middle-income country
PACK: Practical Approach to Care Kit
PHC: Primary Health Care
RtHB: Road to Health Booklet
TB: Tuberculosis
WHO: World Health Organisation

## References

[1] World Health Organisation. Every Woman Every Child. Global strategy for women’s, children’s and adolescents’ health (2016–2030). New York; 2015.

[2] United Nations (2015). Transforming our world: the 2030 Agenda for Sustainable Development.

[3] Liu Li, Oza Shefali, Hogan Dan, Chu Yue, Perin Jamie, Zhu Jun, Lawn Joy E, Cousens Simon, Mathers Colin, Black Robert E (2016). Global, regional, and national causes of under-5 mortality in 2000–15: an updated systematic analysis with implications for the Sustainable Development Goals. The Lancet.

[4] Bamford L J, McKerrow N H, Barron P, Aung Y (2018). Child mortality in South Africa: Fewer deaths, but better data are needed. South African Medical Journal.

[5] The Global Burden of Disease Child and Adolescent Health Collaboration (2017). Child and adolescent health from 1990 to 2015: findings from the global burden of diseases, injuries, and risk factors 2015 study. JAMA Pediatr.

[6] Masekela R, Gray CL, Green J, Manjra AI, Kritzinger FE, Levin M, Zar H. The increasing burden of asthma in South African children: A call to action. South Afr Med J. 2018;108:7.

[7] Haskins L, Grant M, Phakathi S, Wilford A, Jama N, Horwood C. Insights into health care seeking behaviour for children in communities in KwaZulu-Natal, South Africa. Afr J Primary Health Care Family Med. 2017;9(1):1–9. 10.4102/phcfm.v9i1.1378 PMC5458572 28582991

[8] Spark du Preez N. Health-seeking behaviour for childhood illnesses in urban South Africa; 2013. https://ethos.bl.uk/OrderDetails.do?uin=uk.bl.ethos.432254. 10.1016/j.socscimed.2013.05.014 23849278

[9] World Health Organisation. Integrated Management of Childhood Illness. chart booklet. 2014. http://www.who.int/maternal_child_adolescent/documents/IMCI_chartbooklet/en/.

[10] Chopra Mickey, Binkin Nancy J, Mason Elizabeth, Wolfheim Cathy (2012). Integrated management of childhood illness: what have we learned and how can it be improved?. Archives of Disease in Childhood.

[11] Ahmed HM, Mitchell M, Hedt B (2010). National implementation of integrated Management of Childhood Illness (IMCI): policy constraints and strategies. Health Policy.

[12] Gera T, Shah D, Garner P, Richardson M, Sachdev HS. Integrated management of childhood illness (IMCI) strategy for children under five. Cochrane Database Syst Rev. 2016;6:CD010123. 10.1002/14651858.CD010123.pub2 PMC4943011 27378094

[13] Fick C (2017). Twenty years of IMCI implementation in South Africa: accelerating impact for the next decade. South Afr Health Rev.

[14] Horwood Christiane, Vermaak Kerry, Rollins Nigel, Haskins Lyn, Nkosi Phumla, Qazi Shamim (2009). An Evaluation of the Quality of IMCI Assessments among IMCI Trained Health Workers in South Africa. PLoS ONE.

[15] Jacobs M, Merson M (2018). Introductory commentary: a strategic review of options for building on lessons learnt from IMCI and iCCM. BMJ.

[16] Picken Sandy, Hannington Juliet, Fairall Lara, Doherty Tanya, Bateman Eric, Richards Mark, Wattrus Camilla, Cornick Ruth (2018). PACK Child: the development of a practical guide to extend the scope of integrated primary care for children and young adolescents. BMJ Global Health.

[17] Fairall L, Cornick R, Bateman E (2018). Empowering frontline providers to deliver universal primary healthcare using the practical approach to care kit. Br Med J.

[18] Cornick Ruth, Picken Sandy, Wattrus Camilla, Awotiwon Ajibola, Carkeek Emma, Hannington Juliet, Spiller Pearl, Bateman Eric, Doherty Tanya, Zwarenstein Merrick, Fairall Lara (2018). The Practical Approach to Care Kit (PACK) guide: developing a clinical decision support tool to simplify, standardise and strengthen primary healthcare delivery. BMJ Global Health.

[19] Simelane Makhosazana Lungile, Georgeu-Pepper Daniella, Ras Christy-Joy, Anderson Lauren, Pascoe Michelle, Faris Gill, Fairall Lara, Cornick Ruth (2018). The Practical Approach to Care Kit (PACK) training programme: scaling up and sustaining support for health workers to improve primary care. BMJ Global Health.

[20] Yau Matthew, Timmerman Venessa, Zwarenstein Merrick, Mayers Pat, Cornick Ruth Vania, Bateman Eric, Fairall Lara (2019). e-PC101: an electronic clinical decision support tool developed in South Africa for primary care in low-income and middle-income countries. BMJ Global Health.

[21] Zwarenstein M., Fairall L. R., Lombard C., Mayers P., Bheekie A., English R. G., Lewin S., Bachmann M. O., Bateman E. (2011). Outreach education for integration of HIV/AIDS care, antiretroviral treatment, and tuberculosis care in primary care clinics in South Africa: PALSA PLUS pragmatic cluster randomised trial. BMJ.

[22] Fairall Lara R., Folb Naomi, Timmerman Venessa, Lombard Carl, Steyn Krisela, Bachmann Max O., Bateman Eric D., Lund Crick, Cornick Ruth, Faris Gill, Gaziano Thomas, Georgeu-Pepper Daniella, Zwarenstein Merrick, Levitt Naomi S. (2016). Educational Outreach with an Integrated Clinical Tool for Nurse-Led Non-communicable Chronic Disease Management in Primary Care in South Africa: A Pragmatic Cluster Randomised Controlled Trial. PLOS Medicine.

[23] Fairall L, Bachmann MO, Lombard C, Timmerman V, Uebel K, Zwarenstein M, Boulle A, Georgeu D, Colvin CJ, Lewin S, Faris G, Cornick R, Draper B, Tshabalala M, Kotze E, van Vuuren C, Steyn D, Chapman R, Bateman E. Task shifting of antiretroviral treatment from doctors to primary-care nurses in South Africa (STRETCH): a pragmatic, parallel, cluster-randomised trial. Lancet. 2012:380(9845). 10.1016/S0140-6736(12)60730-2 PMC3442223 22901955

[24] Tsima BM, Setlhare V, Nkomazana O. Developing the Botswana primary care guideline: an integrated, symptom-based primary care guideline for the adult patient in a resource-limited setting. J Multidiscip Healthc. 2016;9. 10.2147/JMDH.S112466 PMC4986912 27570457

[25] Wattrus Camilla, Zepeda Jorge, Cornick Ruth Vania, Zonta Ronaldo, Pacheco de Andrade Matheus, Fairall Lara, Georgeu-Pepper Daniella, Anderson Lauren, Eastman Tracy, Bateman Eric D, CRUZ Alvaro A, Bachmann Max O, Natal Sonia, Doherty Tanya, Stelmach Rafael (2018). Using a mentorship model to localise the Practical Approach to Care Kit (PACK): from South Africa to Brazil. BMJ Global Health.

[26] Awotiwon Ajibola, Sword Charlie, Eastman Tracy, Ras Christy Joy, Ana Prince, Cornick Ruth Vania, Fairall Lara, Bateman Eric, Dube Audry, Curran Robyn, Udoekwere Inemesit, Essien Unyime-Obong, Assem Okorie, Edu Theresa Sylvester, Ismail Hajia Binta, Olubajo Olalekan Olugbenga, Ana Joseph (2018). Using a mentorship model to localise the Practical Approach to Care Kit (PACK): from South Africa to Nigeria. BMJ Global Health.

[27] Mekonnen Yibeltal, Hanlon Charlotte, Emyu Solomon, Cornick Ruth Vania, Fairall Lara, Gebremichael Daniel, Teka Telahun, Shiferaw Solomon, Walelgne Wubaye, Mamo Yoseph, Ayehu Temesgen, Wale Meseret, Eastman Tracy, Awotiwon Ajibola, Wattrus Camilla, Picken Sandy Claire, Ras Christy-Joy, Anderson Lauren, Doherty Tanya, Prince Martin James, Tegabu Desalegn (2018). Using a mentorship model to localise the Practical Approach to Care Kit (PACK): from South Africa to Ethiopia. BMJ Global Health.

[28] Chopra M (2005). Effect of an IMCI intervention on quality of care across four districts in Cape Town, South Africa. Archives of Disease in Childhood.

[29] Thandrayen K, Saloojee H (2010). Quality of care offered to children attending primary health care clinics in Johannesburg. South Afr J Child Health.

[30] Sealey Alison (2007). Linguistic ethnography in realist perspective1. Journal of Sociolinguistics.

[31] Rampton BK, Maybin TK, Barwell J, Creese RA, Lytra V (2004). UK linguistic ethnography: a discussion paper.

[32] Murdoch J (2016). Process evaluation for complex interventions in health services research: analysing context, text trajectories and disruptions. BMC Health Serv Res.

[33] Bronfenbrenner U. The ecology of human development. Cambridge: Harvard University Pressl; 1979.

[34] Fryatt R, Hunter J (2014). The ideal Clinic in South Africa: planning for implementation. South Afr Health Rev.

[35] Naidoo H, Avenant T, Goga A (2018). Completeness of the Road-to-Health Booklet and Road-to-Health Card: Results of cross-sectional surveillance at a provincial tertiary hospital. Southern Afr J HIV Med.

[36] Qaseem A, Forland F, Macbeth F, Ollenschlager G (2012). Phillips S, van der Wees. Guidelines International Network: toward international standards for clinical practice guidelines. Annals Internal Med..

[37] Schünemann HJ, Wiercioch W, Etxeandia I, Falavigna M, Santesso N, Mustafa R, et al. Guidelines 2.0: systematic development of a comprehensive checklist for a successful guideline enterprise. 2014;186(3):E123–E42. 10.1503/cmaj.131237 PMC3928232 24344144

[38] Western Cape Government. First 1000 Days Initiative. 2020. https://www.westerncape.gov.za/first-1000-days/. Accessed 20 Feb 2020.

[39] World Health Organization, United Nations Children’s Fund, World Bank Group. Nurturing care for early childhood development: a framework for helping children survive and thrive to transform health and human potential. Geneva: World Health Organization; 2018.

[40] Mitchell JC. Typicality and the case study. Ethnographic research: A guide to general conduct, vol. 238241; 1984.

[41] Stivers T (2005). Parent resistance to physicians' treatment recommendations: one resource for initiating a negotiation of the treatment decision. Health Commun..

[42] Jefferson G (2004). Glossary of transcript symbols with an introduction. Pragmatics and Beyond New Series.

[43] National Department of Health (2014). Standard Treatment Guidelines and Essential Medicines List for South Africa.

[44] National Department of Health. Expanded Programme on Immunization in South Africa (EPI-SA) (2015), (4^th^ Edition). https://www.westerncape.gov.za/assets/departments/health/vaccinators_manual_2016.pdf. Accessed 20 Feb 2020.

[45] Stivers Tanya, Enfield N.J. (2010). A coding scheme for question–response sequences in conversation. Journal of Pragmatics.

[46] Heritage J, Freed S (2009). Questioning in medicine. Why do you ask?: the function of questions in institutional discourse.

[47] Kalu N, Lufesi N, Mortimer K (2016). Implementation of World Health Organization integrated Management of Childhood Illnesses (IMCI) guidelines for the assessment of pneumonia in the under 5s in rural Malawi. PLoS One.

[48] El-Ayady AA, Meleis DE, Ahmed MM, Ismaiel RS (2016). Primary health care physicians’ adherence and attitude towards integrated Management of Childhood Illness guidelines in Alexandria governorate in Egypt. Glob J Health Sci.

[49] Pandya Himani, Slemming Wiedaad, Saloojee Haroon (2017). Health system factors affecting implementation of integrated management of childhood illness (IMCI): qualitative insights from a South African province. Health Policy and Planning.

[50] Gray A, Vawda Y. Health Policy and Legislation. In: Padarath A, King J, Mackie E, Casciola J, editors. South African Health Review 2016. Durban: Health Systems Trust; 2016.

[51] Kagee A., Tsai A. C., Lund C., Tomlinson M. (2012). Screening for common mental disorders in low resource settings: reasons for caution and a way forward. International Health.

