# Supplementary material for: Addressing the quality and scope of paediatric primary care in South Africa: evaluating contextual impacts of the introduction of the Practical Approach to Care Kit for children (PACK Child)
Source: BMC Health Serv Res. 2020 May 29;20:479. doi: 10.1186/s12913-020-05201-w (PMC7257217; doi:10.1186/s12913-020-05201-w)
Supplement: Supplementary file 1 — Additional file 1. Child > 2 months old: Routine Care. Sample of routine care page from PACK Child guide. [file 12913_2020_5201_MOESM1_ESM.pdf]

Child ≥ 2 months old: routine care

Record problems and plot growth in notes and Road to Health Booklet (RtHB).

Assess	When to assess	Note
Symptoms	If sick visit	Manage symptoms on symptom page ↩contents. If child is seriously unwell, manage symptom first.
Feeding	Every visit if < 2 years old	Determine method of feeding. Ask carer if feeding problem. If yes, assess and manage further: if breastfeeding (or mixed feeding) ↩87, if formula feeding ↩89, if eating solids ↩90.
Growth	Check chart ↩14	Interpret measurements ↩15. If born premature, use corrected age[1] until 2 years.
Development *(Screen at every visit. Also check routine milestones at specific ages listed.)*	Every visit	Ask "Is child able to say and do what children of the same age can?" If no, manage problem: if vision problem ↩44, if communication problem ↩81, if not moving or sitting properly ↩82.
	14 weeks old	If unable to follow a close object with eyes ↩44. If does not respond (stops sucking, blinks or turns) to sound ↩81. If unable to lift head when held against shoulder ↩82.
	6 months old	If unable to recognise familiar faces ↩44. If does not turn to look for sound ↩81. If unable to hold a toy in each hand ↩82.
	9 months old	If unable to focus on a far object or has a squint ↩44. If does not turn when called ↩81. If unable to sit and play without support ↩82.
	15 months old	If unable to stand on his/her own ↩82.
	18 months old	If not looking at or reaching for small objects or pictures ↩44. If unable to point to 3 simple objects, uses < 3 words, does not obey simple commands ↩81. If unable to walk unsupported or if unable to feed using fingers ↩82.
	3 years old	If unable to see small shapes clearly from 6 metres ↩44. If unable to talk in simple 3-word sentences ↩81. If unable to run or climb ↩82.
	5 years old	If any problem with vision ↩44. If unable to speak in full sentences or not interacting with children and adults ↩81. If unable to hop on one foot or draw a stick person ↩82.
Well child visits	Every visit	Check if immunisations, deworming, vitamin A are up to date in RtHB and what is due at this visit ↩14. If missed doses, catch up ↩13.
HIV	Every visit if not known HIV positive	• If HIV status unknown, decide if HIV test is needed ↩105.<br>• If HIV negative and breastfeeding, check that mother tests for HIV every 3 months.<br>• If HIV-exposed (mother HIV positive), check child has had routine HIV tests ↩105. Ensure the HIV-exposed baby is receiving PMTCT ↩111.<br>• If HIV positive, ensure on ART and give routine HIV care ↩106.
TB	Every visit	If close TB contact ↩98. If TB symptoms (cough or fever ≥ 2 weeks, not growing well/losing weight, tired/less playful) ↩100.
Mother/carer	Every visit	Ask about general health, HIV status, contraceptive needs and TB symptoms ↩PACK Adult.
Psychosocial risk	Every visit	• If child support grant needed, advise to take child's birth certificate and carer's ID to SASSA[2] to apply.<br>• Look for increased psychosocial risk (carer/parent < 20 years old, family/relationship problems, violence at home, lack of partner/family support, financial difficulty, difficult life event in last year, foreigner): give additional support, review more often if needed and if relevant, link with support services/helpline ↩134.<br>• Screen for depression in carer: in the past month, has carer: 1) felt down, depressed, hopeless or 2) felt little interest or pleasure in doing things? If yes to either ↩PACK Adult.<br>• If yes to both of the following ↩85: 1) Are you struggling with or feeling overwhelmed by parenting? 2) Would you like help with this?<br>• If abuse or neglect suspected ↩78.
Mental health	Every visit	If over past few months, child has been miserable, stressed or angry ↩79 or if problematic change in behaviour ↩80.
School problems	If ≥ 6 years old: every visit	• Check if child at school: if not enrolled in school, refer to social worker.<br>• If poor attendance, bullying, learning problems, difficulty socialising at school ↩83.
Basic examination	Every visit	Check for obvious problems (if < 2 years old, undress child fully): pallor[3] ↩42, skin problem (especially nappy area) ↩67, injury ↩32, if deformity, discuss/refer.

Continue to advise and provide routine care treatment → 13.

[1]Corrected age = actual age in months (or weeks) - number of months (or weeks) premature. To calculate corrected age of 9 month old baby, born premature at 32 weeks (this is 8 weeks or 2 months premature): 9 months - 2 months = 7 months. [2]South Africa Social Security Agency. [3]Look for palmar pallor: child's palm is much less pink than your own. Also look for conjunctival pallor: look for paleness of the lower inner eyelid.
