# Supplementary material for: Addressing the quality and scope of paediatric primary care in South Africa: evaluating contextual impacts of the introduction of the Practical Approach to Care Kit for children (PACK Child)
Source: BMC Health Serv Res. 2020 May 29;20:479. doi: 10.1186/s12913-020-05201-w (PMC7257217; doi:10.1186/s12913-020-05201-w)
Supplement: Supplementary file 2 — Additional file 2. Sick Child Age 2 months to 5 years. Sample of IMCI Checklist. [file 12913_2020_5201_MOESM2_ESM.pdf]

Western Cape Government
Health

BETTER TOGETHER

SICK CHILD AGE 2 MONTHS UP TO 5 YEARS

DATE: ____________ **TIME IN:** ________

Name: ______________________________ DOB: ____________ (dd/mm/yy) Age: _____Mth Weight: _______kg Temp: _____°C

ASK: What are the child's problems? ____ ☐ Initial Visit ☐ Follow up Visit

ASSESS (Mark if present)

**CHECK FOR GENERAL DANGER SIGNS**<br>☐ **NOT ABLE TO DRINK OR BREASTFEED** ☐ **LETHARGIC OR UNCONSCIOUS**<br>☐ **VOMITS EVERYTHING**<br>☐ **CONVULSIONS THIS ILLNESS**	**Danger sign?** ☐**Yes** ☐**No**<br>Use to select classifications
**COUGH OR DIFFICULT BREATHING?** ☐ **Yes** ☐ **No**<br>For how long?_____ days Counted _____ breaths per minute: ☐ Fast breathing<br>☐ Chest indrawing ☐ Stridor ☐ Wheeze<br>**If wheeze ask:**<br>☐ Wheeze before this illness ☐ Frequent cough at night<br>☐ Wheeze for more than 7 days ☐ Treatment for asthma at present	•
**DIARRHOEA?** ☐ **Yes** ☐ **No**<br>For how long? _____ days General condition:<br>☐ Blood in the stool ☐ Lethargic or unconscious ☐ Restless or irritable<br>How much / what fluid mother has given: ☐ Sunken eyes<br>☐ Not able to drink, or drinking poorly<br>☐ Drinking eagerly, thirsty<br>Pinched abdomen skin goes back: ☐ Normally ☐ Slowly ☐ Very slowly (> 2 secs)	•<br>•<br>•
**FEVER (by history or feel or 37.5 ° C or above) ?** ☐ **Yes** ☐ **No**<br>Fever for how long? _____ days ☐ Stiff neck ☐ Red Throat ☐ Bulging fontanelle<br>☐ Malaria Risk. **If malaria risk:** Malaria Test: ☐Positive ☐Negative ☐Not done<br>☐ Cold with runny nose, or other adequate cause of fever<br>☐ Measles rash ☐Red eyes ☐Cornea clouded ☐Deep mouth ulcers ☐Mouth ulcers ☐ Eyes draining pus	
**EAR PROBLEM?** ☐ **Yes** ☐ **No**<br>☐ Ear pain ☐ Wakes child at night? ☐ Pus seen draining from ear<br>☐ Ear discharge reported: for _____ days ☐ Tender swelling behind the ear	
**CHECK FOR MALNUTRITION AND ANAEMIA** **All children**<br>Plot weight for age on the RTH card: ☐ Normal weight ☐ Low weight ☐ Very low weight<br>Join the dots to see weight gain: ☐ Good gain ☐ Poor gain ☐ Losing weight<br>☐ Mother says child 'lost weight' ☐ Oedema of both feet ☐ Visible severe wasting<br>☐ No pallor ☐ Some pallor ☐ Severe pallor<br>**If pale,** Haemoglobin measured _________ gm / dl	ALWAYS classify:<br>•<br>•
**CONSIDER HIV INFECTION** **All children**<br>Has the child had an HIV test? If yes, what was the result? ☐ Pos HIV test ☐ Neg HIV test<br>If test Positive: is child on ART ☐ Yes ☐ No If test negative: ask about breastfeeding<br>If no test, has the mother had an HIV test? ☐ No test ☐ Pos HIV test ☐ Neg HIV test<br>**And:**<br>☐ Pneumonia now ☐ Parotid enlargement<br>☐ Low weight for age ☐ Persistent diarrhoea now or in the past 3 months<br>☐ Poor weight gain ☐ Ear discharge now or in the past<br>☐ Oral thrush ☐ Enlarged glands in 2 or more of: neck, axilla or groin	ALWAYS classify:<br>•<br>•
**CONSIDER TB Does the child have a close TB contact?** ☐ **Yes** ☐ **No**<br>**If yes, or if the child has cough for 2 weeks, is NOT GROWING WELL or has fever for more than 7 days**<br>☐ Persistent cough ☐ Loss of weight ☐ Fatigue ☐ Fever daily for 14 days ☐ On TB Rx	
**CHECK IMMUNIZATION STATUS** **All children**<br>**Circle immunizations given, tick immunizations to be given today**<br>**Birth** ☐ BCG ☐ OPV 0<br>**6 weeks** ☐ DaPT-Hib-IPV 1 ☐ OPV 1 ☐ HepB 1 ☐ PCV 1 ☐ RV 1<br>**10 weeks** ☐ DaPT-Hib-IPV 2 ☐ HepB 2<br>**And** **14 weeks** ☐ DaPT-Hib-IPV 3 ☐ HepB 3 ☐ PCV 2 ☐ RV 2<br>☐ For Vit A today **9 months** ☐ Measles 1 ☐ PCV 3<br>☐ For de-worming today **18 months** ☐ DaPT-Hib-IPV 4 ☐ Measles 2<br>**6 years** ☐ Td	Dosis needed today:<br>Next immunization date:
**ASSESS CHILD'S FEEDING** **if anemia, not growing well or age under two**<br>How are you feeding your child? ______________________<br>☐ Breast fed: ______ times during the day. ☐ Breast fed during the night<br>☐ Given other milk: ____________ type. Using ____________ to give the milk.<br>Other milk given _______ times per day. Amounts of other milk each time: ____________<br>☐ Given other food or fluids. These are: ______________________<br>These given _________ per day. Using ____________ to give other fluids.<br>☐ Feeding changed in this illness. If yes, How? ______________________<br>**If not growing well: How large are the servings?** ______________________<br>☐ Own serving given. Who feeds the child and how?	Feeding problems found:
**ASSESS OTHER PROBLEMS:**	
**ASK ABOUT MOTHER'S OWN HEALTH: FP** ☐ **HIV** ☐ **Cervical Smear** ☐	

Western Cape Government
Health
BETTER TOGETHER.

TREAT THE CHILD

Refer any child who has a danger sign, even if no other severe classification.

ALLERGIES: ______________________________

____
____
____
____
____
____
____
____
____
____
____
____
____
____

Return for follow-up: ______________________________

Advise mother when to return immediately. ______________________________

Give any immunizations needed today: ______________________________

Give routine Vitamin A if needed today: ______________________________

Give worm treatment if needed today: ______________________________

Feeding advice: ______________________________

____
____
____
____
____
____

NB. Make sure of the following before the mother leave the facility: Did you check for Vit A , D worm, Immunisation, HIV, TB

Time Out: ______________________________

Signature: ______________________________
