# Supplementary material for: Addressing the quality and scope of paediatric primary care in South Africa: evaluating contextual impacts of the introduction of the Practical Approach to Care Kit for children (PACK Child)
Source: BMC Health Serv Res. 2020 May 29;20:479. doi: 10.1186/s12913-020-05201-w (PMC7257217; doi:10.1186/s12913-020-05201-w)
Supplement: Supplementary file 3 — Additional file 3. Integrated Stationery for children < 5 years. Sample of Integrated Clinical Stationery used for children less than 5. [file 12913_2020_5201_MOESM3_ESM.pdf]

Date

Notes

*Enter results of annual/frequent tests, drug changes, hospitalisations or observations for monitoring. Insert own headings if needed.*

# INTEGRATED STATIONERY FOR CHILDREN ≤5 YEARS

Western Cape Government    v.201902

## PATIENT DETAILS

| | |
|---|---|
| First name | |
| Surname | |
| DOB | / / Sex M / F |
| ID Number | |

**Address:**

**Caregiver(s) name:**

Tel.: Tel.:

Other contact name: Tel.:

## ALLERGIES / ADVERSE DRUG REACTIONS

**Emergency contact name:**

Tel.: Tel.:

**ECD centre | School | Care** *(circle one and write name below in Facility name field)*

**Facility name:** Tel.:

## Family / social context

**Caregiver(s):** Mother | Mother&Father | Other details

**Caregiver(s) name(s):**

**Caregiver(s) address:**

**Caregiver(s) health status** Healthy | Unwell

**Caregiver(s) risks** Teen Mom | Substances | Other details

**Caregiver income** Salary | Casual | Grant | Other details

**No. children in HH/ premises:** **≤ 5yrs** number | **>5 to 18 yrs** number

**Housing** Formal | Informal details **Electricity** Y | N

**Water supply** Piped inside | Piped outside | Not piped

**Sanitation** Flush toilet | Bucket | Communal

## Birth information

**Weight** kg **Length** cm **HC** cm **Apgar**

**Delivery mode** NVD | C/S | BBA

**Delivery facility**

**Mother HIV+ in labour** Y | N | Ukn **Birth PCR** Y | N | N/A result

**ART prophylaxis** NVP | AZT **TB exposed** Y | N

**Neonatal feeding** EBF | EFF | Mixed

**At risk neonate** Twin | LBW | Prem | Hospital Admission | | Other details

Notes

| Immunisations | | | | | HTS | Developmental screen | Vit A | Deworm | Oral Health |
|---|---|---|---|---|---|---|---|---|---|
| Age group | Date | Vaccine | Site* | Batch no. | Date performed | Date performed | Date performed | Date performed | Date performed |
| Birth | | BCG | R arm | | | | | | |
| | | OPV0 | Oral | | | | | | |
| 6 w | | OPV1 | Oral | | | | | | |
| | | RV1 | Oral | | | | | | |
| | | PCV1 | R thigh | | | | | | |
| | | Hexaxim1 | L thigh | | | | | | |
| 10 w | | Hexaxim2 | L thigh | | | | | | |
| 14 w | | RV2 | Oral | | | | | | |
| | | PCV2 | R thigh | | | | | | |
| | | Hexaxim3 | L thigh | | | | | | |
| 18 w | | | | | high risk | | | | |
| 6 m | | Measles 1 | L thigh | | | | | | |
| 9 m | | PCV3 | R thigh | | | | | | |
| 12 m | | Measles 2 | R arm | | | | | | |
| 18 m | | Hexaxim4 | L arm | | | | | | |
| 2 yrs | | | | | | | | | |
| 2 yrs 6m | | | | | | | | | |
| 3 yrs | | | | | | | | | |
| 3 yrs 6 m | | | | | | | | | |
| 4 yrs | | | | | | | | | |
| 4 yrs 6m | | | | | | | | | |
| 5 yrs | | | | | | | | | |
| 6 wks post cess. BF | | | | | | | | | |

** If child R Handed then L arm. If child L Handed then R arm.*

*Enter details on longterm health conditions, medical, surgical and social history overpage*

Western Cape Government

# LONG TERM HEALTH CONDITIONS AND NOTES

*(for date diagnosed of other conditions, detailed social history and life course information)*

☐ Baseline completed    Date of baseline completion: dd/mm/yyyy

**Diagnosis**

| | | | | | | |
|---|---|---|---|---|---|---|
| ☐ Developmental | dd/mm/yyyy | details | ☐ HIV | Diagnosis | dd/mm/yyy | baseline CD4 |
| ☐ Disability | dd/mm/yyyy | details | | Baseline staging | 1 2 3 4 | |
| ☐ Congenital | dd/mm/yyyy | details | ☐ ART | Prior ART | dd/mm/yyy | drug(s) |
| ☐ Epilepsy | dd/mm/yyyy | details | | Start | dd/mm/yyy | drug(s) |
| ☐ Asthma | dd/mm/yyyy | details | | TFI | dd/mm/yyy | Transferred from |
| ☐ Rhinitis | dd/mm/yyyy | details | | Restart/ change | dd/mm/yyyy | drug(s) |
| ☐ Eczema | dd/mm/yyyy | details | ☐ IPT | Start | dd/mm/yyy | |
| ☐ Allergic Conjuctivitis | dd/mm/yyyy | details | ☐ TB Rx | 1st Treatment | dd/mm/yyy | |
| | | | | ☐ TB Outcome | ☐ DSTB | ☐ DRTB |
| ☐ Other | dd/mm/yyyy | details | | Cured \| Complete \| Default \| Failure | | |
| ☐ Other | dd/mm/yyyy | details | | 2nd Treatment | dd/mm/yyy | |
| ☐ Other | dd/mm/yyyy | details | | ☐ TB Outcome | ☐ DSTB | ☐ DRTB |
| | | | | Cured \| Complete \| Default \| Failure | | |

**Additional notes on medical/surgical/social history:**

STICKER OF MOTHER

---

## Observations and Screening

| | | | |
|---|---|---|---|
| **Date:** dd / mm / yyyy | **Time of initial assessment:** | Emergency Y N | Triage Colour |
| Age: (wk / months / years) | Who brought child in: | Principal caregiver: Mother \| Other | |
| **Ask reasons for visit** details | Well \| Sick \| Trauma | **If sick ≤2m use IMCI stationery** | |
| Danger signs: <2m old \| consult in past 2days \| lethargic or unconscious \| fever; vomiting \| convulsions \| not feeding \| cough \| difficulty breathing \| diarrhoea \| weakness | | Action taken Y N N/A; details | |
| Temp: | RR: | HR: | Sats: |
| Pallor Y N | HB: | Action taken Y N N/A; details | |
| Wt (kg) | Ht/ Length (cm) | MUAC (cm) | HC (cm) |
| Feeding: Age <6m \| ≥6m | EBF \| EFF \| BF \| FF \| Solids | Problem Y N | details |
| Growth: Norm \| OW \| Faltering \| MAM \| SAM \| Catch up \| Stunted | Devel: Norm \| Problem | details | |
| Immun: UTD \| Due | Deworm: UTD \| Due | Vit A: UTD \| Due | Oral health: Norm \| Problem; details |
| On TB Rx Y N | TB Rx duration (months) | TB Screen: Cough \| TB Contact \| Fever \| Not growing well \| Fatigue or playing less | |
| HIV status: Known pos \| Neg last test \| Unkn | Next HIV test due: Today \| Other dd/mm/yyyy | On IPT Y N N/A | |
| Duration on current ART: (months) | Next VL test due: date | On Bactrim Y N N/A | Duration on IPT months |

## Care-giver

| | | | |
|---|---|---|---|
| **Mother:** Well Y N N/A | HIV: Known pos \| Neg last test \| Test due | FP: UTD \| Due type | Condoms used Y N N/A |
| **Caregiver:** Mother \| Other | TB Y N | Substance abuse Y N | Psychosocial risk Y N details |

## History and Exam

**Time of consultation:**    **Routine care:** Immunise \| Vit A \| Deworm

**Other IMCI:** Measles \| Ear problems \| Sore throat \| Other

## Assessment and Management

| Assessment / Diagnosis / Problem List | Management / Medication / Supplements |
|---|---|
| 1 | |
| 2 | |
| 3 | |
| 4 | |
| **Referral** Y N details | ART med(s) change: Y N; ART prescribed; Duration Prescribed |

**Counselling/ Health Education:** details / record on counselling stationery    Updated RTHB Y N

Counselling given for: HIV test \| Other procedure type Y N    Consent given: Y N    Name:    Signature:    Parent \| Caregiver

## Data Elements

**CHILD:** ☐ HIV Test    POS \| NEG (screening)    POS \| NEG (confirmatory)    **Write test details overpage**    **MOM:** ☐ Rapid HIV Test    POS \| NEG (screening)    POS \| NEG (confirmatory)

☐ VL result    ☐ TST POS \| NEG    ☐ X-rays Norm \| Abn (enter details on back of outer)

☐ Urine dipstix result    ☐ Urine MC & S result

☐ Other test: type result    ☐ Other test: type result    ☐ Other test: type result

| | | | | | | | | | | | | |
|---|---|---|---|---|---|---|---|---|---|---|---|---|
| BCG | RV1 | OPV1 | HX1 | PCV1 | HX2 | RV2 | PCV2 | HX3 | M1 | PCV3 | HX4 | M2 |
| FImm | Vit A | DeW | EBF | EBF14 | MAM | SAM | FdSup | TD1 | TD2 | HPV1 | HPV2 | Other |
| DD | DND | Pneu | ASTHv | HIVv | TBv | TBs | TBsHIV | TBcASx | TBSx | IPTeHIV | TBcIPTi | IPTiHIV |
| Appt | Ref | Disch. | OHv | EPILv | MHv | Allied health | Allied health | Allied health | Allied health | Other | Other | Other |

| | | | | | |
|---|---|---|---|---|---|
| PN | DR | OHP | Allied HP | Allied profession | Other: Job type/title |

Reason(s) for next visit:

v.201905

Name:

SANC/HPCSA no.    STAMP

Signature:

Date of next visit:

Date captured:

Clerk (sign):

Western Cape Government

# Notes to guide completion of child stationery

## For visit summary

1. For 'emergency' patients, complete the top line (Y or N and triage colour). Write 'refer to Triage notes' in the History and Examination section and use separate triage stationery.
2. If extra space is required to record a visit, clearly mark PTO in the History/Examination block; on the space allocated at the back of the page, clearly mark the date of these extra notes.
3. Where there are several choices, circle all that apply e.g. for feeding, one might circle 'FF' and 'Solids'.
4. Stippled blocks indicate IMCI classifications e.g. danger signs and feeding assessment in the top section of visit summary, and 'Other IMCI' at the bottom of History and examination section.
5. Growth measurements are recorded in the visit summary, then plotted in the RTHB, and then interpreted in the visit summary.
6. Development must be assessed as Normal/problem using the assessment questions in the RTHB.
7. For Assessment and management section:
   a. record an assessment/diagnosis and the management thereof on the same numbered line. For a chronic/ongoing condition, it is advisable to keep that condition on the same line at every visit.
   b. Medications may be summarized in this section, as the full details will be on the prescription chart.
   c. For ART, it is necessary (for data capturing purposes) to indicate the names of the ART prescribed at the visit, whether these have been changed at that visit, and the duration for which the ART has been prescribed.
8. Details of counselling/health education could be entered in the separate counselling stationery.
9. For HIV testing: consent for testing, and the results of the test are recorded in the visit summary. The details of the actual screening and confirmatory tests (names, batch no., expiry date) are recorded on the section at the back of the visit summary.
10. Tests: tick the box associated with the test that is to be performed at that visit. When the result is received, record the result next to this ticked box i.e. in the record for the visit at which the test was taken.
11. For sites where electronic capturing of RMR/other data is occurring: put a cross through the shaded block(s) which apply to that visit. For allied health professionals, enter t/he abbreviation of the relevant RMR element into one of the open boxes allocated for this and mark a cross over the element. In cases where a number is also required, include this. For example: Tex2 indicates 2 teeth extracted.
12. Putting a cross through a shaded date element for routine care indicates that the clinician has administered that treatment/prevention measure e.g. crossing through M1 indicates that the first measles immunistaion has been given. This action need not then be recorded again in the visit notes.
13. If routine care is given, the table on the front of the outer must be updated.
14. The RTHB must be updated at every visit.
15. At the end of every visit, consider whether any updating of the patient summary (the 'outer') is required.

## Data elements: RMR and other

| | |
|---|---|
| BCG | BCG |
| HX 1 2 3 4 | Hexaxim 1-4 |
| PCV 1 2 3 | Pneumococcal vaccine 1-3 |
| RV 1 2 | Rotavirus 1-2 |
| OPV 1 | Oral Polio 1 |
| M1 2 | Measles 1-2 |
| FIMM | Fully immunised (under one year) |
| TD1 2 | Tetanus 1-2 |
| HPV 1 2 | HPV 1-2 |
| VitA | Vitamin A |
| DeW | Deworm |
| EBF | Exclusively breastfed |
| EBF14 | Exclusively breastfed at 14 weeks |
| MAM | Moderate acute malnutrition (new) |
| SAM | Severe acute malnutrition (new) |
| FdSup | Food supplements given (new) |
| DD | Diarrhoea with dehydration (new episode) |
| DND | Diarrhoea with no dehydration |
| Pneu | Pneumonia (new episode) |

**Other**:

| | |
|---|---|
| HIVv | Visit of a client with HIV |
| ASTHv | Visit of a client with Asthma |
| EPILv | Visit of a client with epilepsy |
| MHv | Mental health visit |
| OHv | Oral health visit |
| Appt | Client given appointment to return |
| Ref | Client referred to another facility/service |
| Disch | Client discharged with no appointment or referral |

**TB elements**:

| | |
|---|---|
| TBs | Screened for TB symptoms |
| TBsHIV | HIV positive client screened for TB |
| TBSx | TB symptoms |
| TBcASx | TB contact but asymptomatic for TB |
| TBcIPTi | TB contact started on IPT |
| TBv | Visit of a client with TB |
| IPTeHIV | Newly diagnosed HIV positive child eligible for IPT |
| IPTi HIV | Newly diagnosed HIV positive child initaited on IPT |

**Allied Health:**
*(use one/more block(s) to write relevant RMR elements)*

| | |
|---|---|
| WCr or Wci | wheelchair required or issued |
| SPr or SPi | spectacles required or issued |
| HAr or HAi | hearing aid required or issued |
| Tex no. | number of teeth extracted |
| Trest no. | number of teeth restored |
| Tfs | tooth fissure sealants 1st and 2nd permanent molar (child) |

| | |
|---|---|
| ART | Antiretroviral treatment |
| AZT | Zidovudine |
| BBA | Born before arrival |
| BF | Breastfed (not exclusively) |
| C/S | Caesarian section |
| Dev. | Development |
| DOB | Date of birth |
| DSTB | Drug sensitive tuberculosis |
| DRTB | Drug resistant tuberculosis |
| EBF | Exclusively breastfeeding |
| ECD | Early childhood development |
| EFF | Exclusive formula feeding |
| FF | Formula fed (not exclusively) |
| FP | Family planning (contraception) |
| HB | Haemaglobin |
| HC | Head circumference |
| HIV | Human immunodeficiency virus |
| HH | Household |
| HR | Heart rate |
| Ht | Height |
| HTS | HIV testing and support |
| IMCI | Integrated management of childhood illnesses |
| Immun | Immunisation |
| IPT | Isoniazid preventive therapy |
| LBW | Low birth weight (<2.5kg) |

| | |
|---|---|
| MAM | Moderately acute malnutrition (see definition) |
| MC+S | Microscopy culture and sensitivity |
| MUAC | Mid upper arm circumference |
| Norm. | Normal |
| NVD | Normal vaginal delivery |
| NVP | Neviripine |
| OHP | Oral health practitioner |
| OW | Overweight |
| PCR | Polymerase chain reaction test (HIV) |
| Prem. | Premature infant (born before 37 weeks) |
| Rif S/R | Rifampicin sensitive or resistant |
| RTHB | Road to health booklet |
| RR | Respitatory rate |
| Rx | Treatment (medication) |
| SAM | Severe acute malnutrition (see definition) |
| Sats | Oxygen saturation |
| TB | Tuberculosis |
| Temp | Temperature |
| TFI | Transferred in |
| TST | Tuberculin skin test (Mantoux) |
| Unkn | Unknown |
| UTD | Up to date |
| Vit A | Vitamin A |
| VL | Viral load (HIV) |
| Wt | Weight |

STICKER

v.201905

__Growth:__ *plot height & weight on growth chart in RTHB & interpret according to definition below. Note, circle multiple categories if needed eg MAM&catch up*

1. __Normal growth:__ weight & height are appropriate for age & follow the normal growth curve (between the -2 to +2 lines). Note: with preterm & LBW infants, growth must follow their respective growth curve, which may be lower than in a full term infant.
2. __Overweight:__ assess weight for age and weight for height / length. If weight for age is above +3SD in the growth chart, plot it on the weight for height / length chart. Classify as overweight (OW) if the weight for height / length is between +2SD and +3SD; classify as obese if weight for height / length is > 3SD.
3. __Growth faltering:__ not gaining weight / flattening of growth curve on growth chart. If < 6 months: flattening over 2 consecutive visits. If aged 6 months – 5 years: flattening of curve over 2 months.
4. __MAM:__ moderate acute malnutrition. Weight for height between -2 & -3 line or MUAC (children 6 months – 5 years) between 11.5 & 12.5 cm.
5. __SAM:__ severe acute malnutrition: 1 of the following: Weight for height < -3 line; MUAC < 11.5 (children 6 - 60 months) or pitting oedema with normal weight.
6. __Catch up:__ growth improving after intervention for faltering growth or SAM / MAM.

## Continuation notes *(please date each entry and only use for visits included on the reverse)*

Date

### HIV Testing

Test date: dd/mm/yyyy *(enter results of tests in visit summary for this test date)*

Name of HIV screening test (PCR / Rapid) ______ Batch no. ______ Expiry Date dd/mm/yyyy

Name of HIV confirmatory test (PCR / Rapid) ______ Batch no. ______ Expiry date dd/mm/yyyy

**Initial Assessment**

| | | | |
|---|---|---|---|
| **Date:** dd / mm / yyyy | **Time of initial assessment:** | Emergency Y N | Triage Colour |
| Age: (wk / months / years) | Who brought child in: | Principal caregiver: Mother \| Other | |
| **Ask reasons for visit** details | Well \| Sick \| Trauma | **If sick ≤2m use IMCI stationery** | |
| Danger signs: <2m old \| consult in past 2days \| lethargic or unconscious \| fever | | Action taken Y N N/A | |
| vomiting \| convulsions \| not feeding \| cough \| difficulty breathing \| diarrhoea \| weakness | | details | |

**Vitals & Measurem**

| | | | | | | |
|---|---|---|---|---|---|---|
| Temp: | RR: | HR: | Sats: | Pallor Y N | HB: | Action taken Y N N/A |
| Wt (kg) | Ht/ Length (cm) | MUAC (cm) | HC (cm) | | | details |

**Routine**

Feeding: Age: <6m | ≥6m EBF | EFF | BF | FF | Solids Problem Y N details

Growth: Norm | OW | Faltering | MAM | SAM | Catch up | Stunted Devel: Norm | Problem details

Immun: UTD | Due Deworm: UTD | Due Vit A: UTD | Due Oral health: Norm | Problem details

On TB Rx Y N TB Rx duration (months) TB Screen: Cough | TB Contact | Fever | Not growing well | Fatigue or playing less

HIV status: Known pos | Neg last test | Unkn Next HIV test due: Today | Other dd/mm/yyyy On IPT Y N N/A

Duration on current ART: (months) Next VL test due: date On Bactrim Y N N/A Duration on IPT months

**Caregiver**

**Mother:** Well Y N N/A HIV: Known pos | Neg last test | Test due FP: UTD | Due type Condoms used Y N N/A

**Caregiver:** Mother | Other TB Y N Substance abuse Y N Psychosocial risk Y N details

**History & Examination**

**Time of consultation:** **Routine care:** Immunise | Vit A | Deworm

**Other IMCI:** Measles | Ear problems | Sore throat | Other

**Assessment & management**

| **Assessment / Diagnosis / Problem List** | **Management / Medication / Supplements** |
|---|---|
| **1** | |
| **2** | |
| **3** | |
| **4** | |

**Referral** Y N details **ART med(s) change:** Y N ART prescribed Duration Prescribed

**Counselling/ Health Education:** details / record on counselling stationery **Updated RTHB** Y N

**Counselling given for** HIV test | Other procedure type Y N **Consent given:** Y N Name: Signature: Parent | Caregiver

**Investigations**

**CHILD:** ☐ HIV Test POS | NEG screening POS | NEG confirmatory **Write test details overpage** **MOM:** ☐ Rapid HIV Test POS | NEG screening POS | NEG confirmatory

☐ VL result ☐ TST POS | NEG ☐ X-rays Norm | Abn (enter details on back of outer)

☐ Urine dipstix result ☐ Urine MC & S result

☐ Other test: type result ☐ Other test: type result ☐ Other test: type result

**Data elements**

| | | | | | | | | | | | | |
|---|---|---|---|---|---|---|---|---|---|---|---|---|
| BCG | RV1 | OPV1 | HX1 | PCV1 | HX2 | RV2 | PCV2 | HX3 | M1 | PCV3 | HX4 | M2 |
| FImm | Vit A | DeW | EBF | EBF14 | MAM | SAM | FdSup | TD1 | TD2 | HPV1 | HPV2 | Other |
| DD | DND | Pneu | ASTHv | HIVv | TBv | TBs | TBsHIV | TBcASx | TBSx | IPTeHIV | TBcIPTi | IPTiHIV |
| Appt | Ref | Disch. | OHv | EPILv | MHv | Allied health | Allied health | Allied health | Allied health | Other | Other | Other |

PN DR OHP Allied HP Allied profession Other: Job type/title Reason(s) for next visit:

Name:

SANC/HPCSA no. STAMP

Signature:

Date of next visit:

Date captured:

Clerk (sign):

v.201905


**Initial Assessment**

| | | | |
|---|---|---|---|
| **Date:** dd / mm / yyyy | **Time of initial assessment:** | Emergency Y N | Triage Colour |
| Age: (wk / months / years) | Who brought child in: | Principal caregiver: Mother \| Other | |
| **Ask reasons for visit** details | Well \| Sick \| Trauma | **If sick ≤2m use IMCI stationery** | |
| Danger signs: <2m old \| consult in past 2days \| lethargic or unconscious \| fever | | Action taken Y N N/A | |
| vomiting \| convulsions \| not feeding \| cough \| difficulty breathing \| diarrhoea \| weakness | | details | |

| | | | | | | |
|---|---|---|---|---|---|---|
| Temp: | RR: | HR: | Sats: | Pallor Y N | HB: | Action taken Y N N/A |
| Wt (kg) | Ht/ Length (cm) | MUAC (cm) | HC (cm) | | | details |

Feeding: Age: <6m | ≥6m EBF | EFF | BF | FF | Solids Problem Y N details

Growth: Norm | OW | Faltering | MAM | SAM | Catch up | Stunted Devel: Norm | Problem details

Immun: UTD | Due Deworm: UTD | Due Vit A: UTD | Due Oral health: Norm | Problem details

On TB Rx Y N TB Rx duration (months) TB Screen: Cough | TB Contact | Fever | Not growing well | Fatigue or playing less

HIV status: Known pos | Neg last test | Unkn Next HIV test due: Today | Other dd/mm/yyyy On IPT Y N N/A

Duration on current ART: (months) Next VL test due: date On Bactrim Y N N/A Duration on IPT months

**Mother:** Well Y N N/A HIV: Known pos | Neg last test | Test due FP: UTD | Due type Condoms used Y N N/A

**Caregiver:** Mother | Other TB Y N Substance abuse Y N Psychosocial risk Y N details

**Time of consultation:** **Routine care:** Immunise | Vit A | Deworm

**Other IMCI:** Measles | Ear problems | Sore throat | Other

| **Assessment / Diagnosis / Problem List** | **Management / Medication / Supplements** |
|---|---|
| **1** | |
| **2** | |
| **3** | |
| **4** | |

**Referral** Y N details **ART med(s) change:** Y N ART prescribed Duration Prescribed

**Counselling/ Health Education:** details / record on counselling stationery **Updated RTHB** Y N

**Counselling given for** HIV test | Other procedure type Y N **Consent given:** Y N Name: Signature: Parent | Caregiver

**CHILD:** ☐ HIV Test POS | NEG screening POS | NEG confirmatory **Write test details overpage** **MOM:** ☐ Rapid HIV Test POS | NEG screening POS | NEG confirmatory

☐ VL result ☐ TST POS | NEG ☐ X-rays Norm | Abn (enter details on back of outer)

☐ Urine dipstix result ☐ Urine MC & S result

☐ Other test: type result ☐ Other test: type result ☐ Other test: type result

| | | | | | | | | | | | | |
|---|---|---|---|---|---|---|---|---|---|---|---|---|
| BCG | RV1 | OPV1 | HX1 | PCV1 | HX2 | RV2 | PCV2 | HX3 | M1 | PCV3 | HX4 | M2 |
| FImm | Vit A | DeW | EBF | EBF14 | MAM | SAM | FdSup | TD1 | TD2 | HPV1 | HPV2 | Other |
| DD | DND | Pneu | ASTHv | HIVv | TBv | TBs | TBsHIV | TBcASx | TBSx | IPTeHIV | TBcIPTi | IPTiHIV |
| Appt | Ref | Disch. | OHv | EPILv | MHv | Allied health | Allied health | Allied health | Allied health | Other | Other | Other |

PN DR OHP Allied HP Allied profession Other: Job type/title Reason(s) for next visit:

Name:

SANC/HPCSA no. STAMP

Signature:

Date of next visit:

Date captured:

Clerk (sign):



**Initial Assessment**

| | | | |
|---|---|---|---|
| **Date:** dd / mm / yyyy | **Time of initial assessment:** | Emergency Y N | Triage Colour |
| Age: (wk / months / years) | Who brought child in: | Principal caregiver: Mother \| Other | |
| **Ask reasons for visit** details | Well \| Sick \| Trauma | **If sick ≤2m use IMCI stationery** | |
| Danger signs: <2m old \| consult in past 2days \| lethargic or unconscious \| fever | | Action taken Y N N/A | |
| vomiting \| convulsions \| not feeding \| cough \| difficulty breathing \| diarrhoea \| weakness | | details | |

| | | | | | | |
|---|---|---|---|---|---|---|
| Temp: | RR: | HR: | Sats: | Pallor Y N | HB: | Action taken Y N N/A |
| Wt (kg) | Ht/ Length (cm) | MUAC (cm) | HC (cm) | | | details |

Feeding: Age: <6m | ≥6m EBF | EFF | BF | FF | Solids Problem Y N details

Growth: Norm | OW | Faltering | MAM | SAM | Catch up | Stunted Devel: Norm | Problem details

Immun: UTD | Due Deworm: UTD | Due Vit A: UTD | Due Oral health: Norm | Problem details

On TB Rx Y N TB Rx duration (months) TB Screen: Cough | TB Contact | Fever | Not growing well | Fatigue or playing less

HIV status: Known pos | Neg last test | Unkn Next HIV test due: Today | Other dd/mm/yyyy On IPT Y N N/A

Duration on current ART: (months) Next VL test due: date On Bactrim Y N N/A Duration on IPT months

**Mother:** Well Y N N/A HIV: Known pos | Neg last test | Test due FP: UTD | Due type Condoms used Y N N/A

**Caregiver:** Mother | Other TB Y N Substance abuse Y N Psychosocial risk Y N details

**Time of consultation:** **Routine care:** Immunise | Vit A | Deworm

**Other IMCI:** Measles | Ear problems | Sore throat | Other

| **Assessment / Diagnosis / Problem List** | **Management / Medication / Supplements** |
|---|---|
| **1** | |
| **2** | |
| **3** | |
| **4** | |

**Referral** Y N details **ART med(s) change:** Y N ART prescribed Duration Prescribed

**Counselling/ Health Education:** details / record on counselling stationery **Updated RTHB** Y N

**Counselling given for** HIV test | Other procedure type Y N **Consent given:** Y N Name: Signature: Parent | Caregiver

**CHILD:** ☐ HIV Test POS | NEG screening POS | NEG confirmatory **Write test details overpage** **MOM:** ☐ Rapid HIV Test POS | NEG screening POS | NEG confirmatory

☐ VL result ☐ TST POS | NEG ☐ X-rays Norm | Abn (enter details on back of outer)

☐ Urine dipstix result ☐ Urine MC & S result

☐ Other test: type result ☐ Other test: type result ☐ Other test: type result

| | | | | | | | | | | | | |
|---|---|---|---|---|---|---|---|---|---|---|---|---|
| BCG | RV1 | OPV1 | HX1 | PCV1 | HX2 | RV2 | PCV2 | HX3 | M1 | PCV3 | HX4 | M2 |
| FImm | Vit A | DeW | EBF | EBF14 | MAM | SAM | FdSup | TD1 | TD2 | HPV1 | HPV2 | Other |
| DD | DND | Pneu | ASTHv | HIVv | TBv | TBs | TBsHIV | TBcASx | TBSx | IPTeHIV | TBcIPTi | IPTiHIV |
| Appt | Ref | Disch. | OHv | EPILv | MHv | Allied health | Allied health | Allied health | Allied health | Other | Other | Other |

PN DR OHP Allied HP Allied profession Other: Job type/title Reason(s) for next visit:

Name:

SANC/HPCSA no. STAMP

Signature:

Date of next visit:

Date captured:

Clerk (sign):
