# Supplementary material for: Addressing the quality and scope of paediatric primary care in South Africa: evaluating contextual impacts of the introduction of the Practical Approach to Care Kit for children (PACK Child)
Source: BMC Health Serv Res. 2020 May 29;20:479. doi: 10.1186/s12913-020-05201-w (PMC7257217; doi:10.1186/s12913-020-05201-w)
Supplement: Supplementary file 4 — Additional file 4. Road to Health Booklet. A sample of the road to health booklet (version used during the pilot). [file 12913_2020_5201_MOESM4_ESM.pdf]

Child under 2 months and:
- is not feeding
- has fever

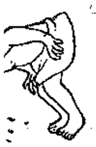

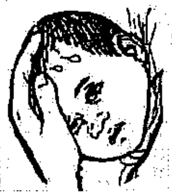

Chest indrawing

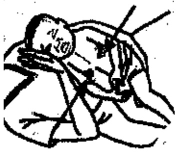

Diarrhoea with sunken eyes or sunken fontanelle

Diarrhoea with blood

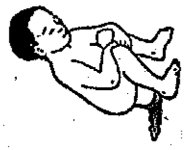

IMPORTANT: Always bring this booklet when you visit any health clinic, doctor or hospital

ROAD TO HEALTH
GIRLS

Child's first name and surname:

____

Date of Birth:

____

This booklet must be issued at birth by the health services concerned.
If birth takes place at home, the first opportunity after delivery should be used to issue the booklet.
The booklet must be issued **FREE OF CHARGE,** irrespective of delivery taking place at a public or private health facility.

health
Department:
Health
REPUBLIC OF SOUTH AFRICA

WELL CHILD VISITS – RECORDING SHEET FOR CHILDREN

Record the following information for each visit on the spaces that are not shaded. Refer to the page numbers given in this booklet and complete the relevant section.						Remember to check the following. Tick if done, and record details on the relevant page					Date of next visit
Age	Date	Growth (IMCI) (page 14)	PMTCT/ HIV status (IMCI) (page 7&8)	TB status (IMCI)	Feeding (EBF/EFF/ mixed feeding for first 6 months)	Immunisations (page 6)	Vitamin A (page 9)	Deworming (page 9)	Development (page 13)	Oral Health (page 20)	
3-6 days											
6 wks											
10 wks											
14 wks											
4 mths											
5 mths											
6 mths											
7 mths											
8 mths											
9 mths											
10 mths											

Age	Date	Growth (IMCI) (page 14)	PMTCT/ HIV status (IMCI) (page 7&8)	TB status (IMCI)	Feeding (EBF/EFF/ mixed feeding for first 6 months)	Immunisations (page 6)	Vitamin A (page 9)	Deworming (page 9)	Development (page 13)	Oral Health (page 20)	Date of next visit
11mths											
12 mths											
14 mths											
16 mths											

)e completed at birth)

:ility where child was born:

th date:

ıe child live with?

luding this child?)

h(s):

ven: / / /
dd mm yyyy

with X)
ıealth services)

ıer need additional
e for the child? Yes No

ıo issued booklet

IMMUNISATIONS

Name and surname:

ID number:

Age group	Batch no.	Vaccine	Site	Date given dd/mm/yy	Signature
Birth		BCG	Right arm		
		OPV0	Oral		
6 weeks		OPV1	Oral		
		RV1	Oral		
		DTaP-IPV-Hib1	Left thigh		
		Hep B1	Right thigh		
		PCV 1	Right thigh		
10 weeks		DTaP-IPV-Hib2	Left thigh		
		Hep B2	Right thigh		
14 weeks		DTaP-IPV-Hib3	Left thigh		
		Hep B3	Right thigh		
		PCV2	Right thigh		
		RV2	Oral		
9 months		Measles1	Left thigh		
		PCV3	Right thigh		
18 months		DTaP-IPV-Hib4	Left arm		
		Measles2	Right arm		
6 years		Td	Left arm		
12 years		Td	Left arm		

HEAD CIRCUMFERENCE AT 14 WEEKS AND AT 12 MONTHS

14 Weeks: ________ (Range: 37 - 42 cm) **12 Months:** _______ (Range: 42 - 47.5)

REFER if head circumference is outside range

ION

Head circumference at birth:

Mother's RPR

(including mode of delivery)

Exclusive formula

lother Care)

natal ward/premature):

PMTCT/HIV INFORMATION

Child's first name and surname:

Child's ID Number: | | | | | | | | | | | | | |

Signature of consent: ____________________

Date:

Fill in this section on discharge from Midwife Obstetric Unit (MOU) or obstetric ward or at first subsequent visit if not yet done

Mother's latest HIV test result	Positive	Negative	To be done
When did mother have the test?	☐ Before pregnancy	☐ During pregnancy	☐ At delivery
Is the mother on life-long ART?	Yes	No	
If yes, duration of life-long ART at time of delivery	☐ < 4 weeks	☐ > 4 weeks	☐ Before pregnancy
Document ARVs the mother received:			
Did the mother receive infant feeding counseling?	Yes	No	
Decision about infant feeding	☐ Exclusive breast	☐ Exclusive formula	
Document Nevirapine given:			

All HIV exposed infants should receive Nevirapine for a minimum of 6 weeks

Has the mother disclosed to anyone in the household?	Yes	No
Has the mother's partner been tested?	Yes	No

Remember to offer testing for all the mother's other children if not yet done

Offer a mother with unknown HIV status a rapid HIV test.

If mother's HIV rapid test is positive, perform an HIV DNA PCR test on infant if ≥ 6/52

HIV exposed

☐ Exclusive formula ☐ Mixed feeding

ıHLS tracking barcoded sticker

Stop now | Continue

ɔr the child has stopped breast-

ıative

levirapine if PCR is positive

Stop now | Continue

ve to continue breastfeeding

ion of breastfeeding, or if clinical

a rapid HIV Antibody test at 18

body test ☐ Positive ☐ Negative

evirapine if PCR is positive

Stop now | Continue

clinical notes ☐

VITAMIN A SUPPLEMENTATION

	At age	Date given dd/mm/yy	Signature	At age	Date given dd/mm/yy	Signature
100 000 IU	6 mths	/ /				
200 000 IU every 6 months	12 mths	/ /		42 mths	/ /	
	18 mths	/ /		48 mths	/ /	
	24 mths	/ /		54 mths	/ /	
	30 mths	/ /		60 mths	/ /	
	36 mths	/ /				

ADDITIONAL DOSES:

For conditions such as measles, severe malnutrition, xerophthalmia and persistent diarrhoea. Omit if dose has been given in last month.
Measles and xerophthalmia: Give one dose daily for two consecutive days. Record the reason and dose given below.

Date	Dose given	Reason	Signature	Date	Dose given	Reason	Signature

DEWORMING TREATMENT (Mebendazole or Albendazole)

Dose	At age	Date given dd/mm/yy	Signature	At age	Date given dd/mm/yy	Signature
	12 mths	/ /		18 mths	/ /	
	24 mths	/ /		48 mths	/ /	
	30 mths	/ /		54 mths	/ /	
	36 mths	/ /		60 mths	/ /	
	42 mths	/ /				

SSAGES

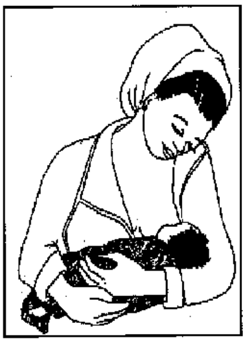

important?

it and make it easy for infections

/, discuss safe preparation

and move.

ile at him or her
sation going with sounds or

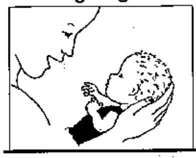

HEALTH PROMOTION MESSAGES

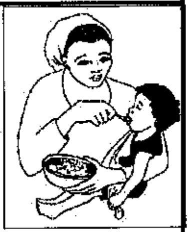

6 - 12 months

Feeding:

For all children start complementary foods at 6 months

- Continue breastfeeding;
- Always breastfeed first before giving complementary foods;
- Start giving 2—3 teaspoons of mashed dried beans and/or locally available animal foods daily to supplement the iron in the breastmilk. Examples include egg (yolk), minced meat, fish, chicken/chicken livers, mopani worms. Give soft porridge, vegetables and then fruit;
- Gradually increase the amount and frequency of feeds.
- Children between 6—8 months should have two meals a day. By 12 months this should have increased to 5 small meals per day, whilst frequent breastfeeding continues;
- Offer your baby safe, clean water regularly;
- If the baby is not breastfed, give formula or at least 2 cups of full cream cow's milk (cow's milk can be given from 9 months of age)

Play: Give your child clean household things to handle, bang and drop.

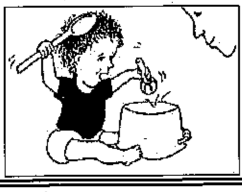

Communicate:

Respond to your child's sounds and interests. Tell your child the names of things and people.

Encourage feeding during illness Suggest an extra meal a day for a week after getting better

Feeding recommendation for DIARRHOEA

- Follow feeding recommendations for the child's age, but give small frequent meals (at least 6 times a day);
- Give a sugar-salt solution (SSS) in addition to feeds. Give SSS after each loose stool, using frequent small sips from a cup (half cup for children under 2 years and 1 cup for children 2—5 years). If the child vomits, wait for 10 minutes then continue, but more slowly

How to prepare a sugar-salt solution (SSS) at home

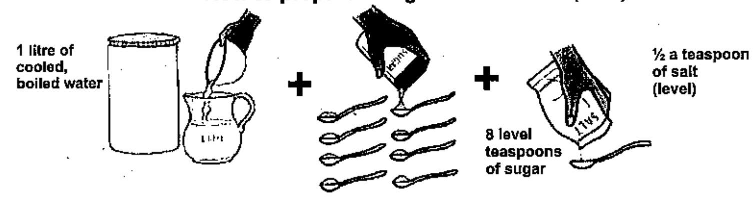

Play and communicate: 12 months to 2 years

Play: Give your child things to stack up, and to put into containers and take out.

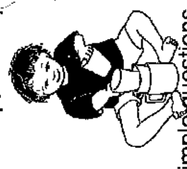

Communicate: Ask your child simple questions. Respond to your child's attempts to talk. Play games like "bye".

Play and communicate: Above 2 years

Play: Help your child count, name, and compare things.

Make simple toys for your child.

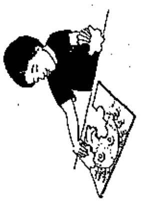

Communicate: Encourage your child to talk and answer your child's questions. Teach your child stories, songs and games.

DEVELOPMENTAL SCREENING

	VISION AND ADAPTIVE	HEARING AND COMMUNICATION	MOTOR DEVELOPMENT
ALWAYS ASK	Can your child see?	Can your child hear and communicate as other children?	Does your child do the the same things as other children of the same age?
14 weeks	Baby follows close objects with eyes	Baby responds to sound by stopping sucking, blinking or turning	Child lifts head when held against shoulder 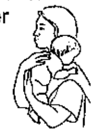
6 months	Baby recognises familiar faces	Child turns head to look for sound	Child holds a toy in each hand 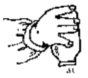
9 months	Child's eyes focus on far objects<br><br>Eyes move well together (No squint)	Child turns when called	Child sits and plays without support 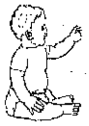
18 months	Child looks at small things and pictures	Child points to 3 simple objects<br><br>Child uses at least 3 words other than names<br><br>Child understands simple commands	Child walks well 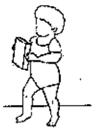<br><br>Child uses fingers to feed
3 years	Sees small shapes clearly at 6 metres	Child speaks in simple 3 word sentences	Child runs well and climbs on things
5-6 years: School readiness	No problem with vision, use a Snellen E chart to check	Speaks in full sentences and interact with children and adults	Hops on one foot 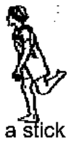<br><br>Able to draw a stick person
REFER	**Refer the child to the next level of care if child has not achieved the developmental milestone. Refer motor problem to Occupational Therapist/Physiotherapist and hearing and speech problem to Speech therapist/Audiologist if you have the services at your facilities.**		

Girl's Weight-for-Age Chart

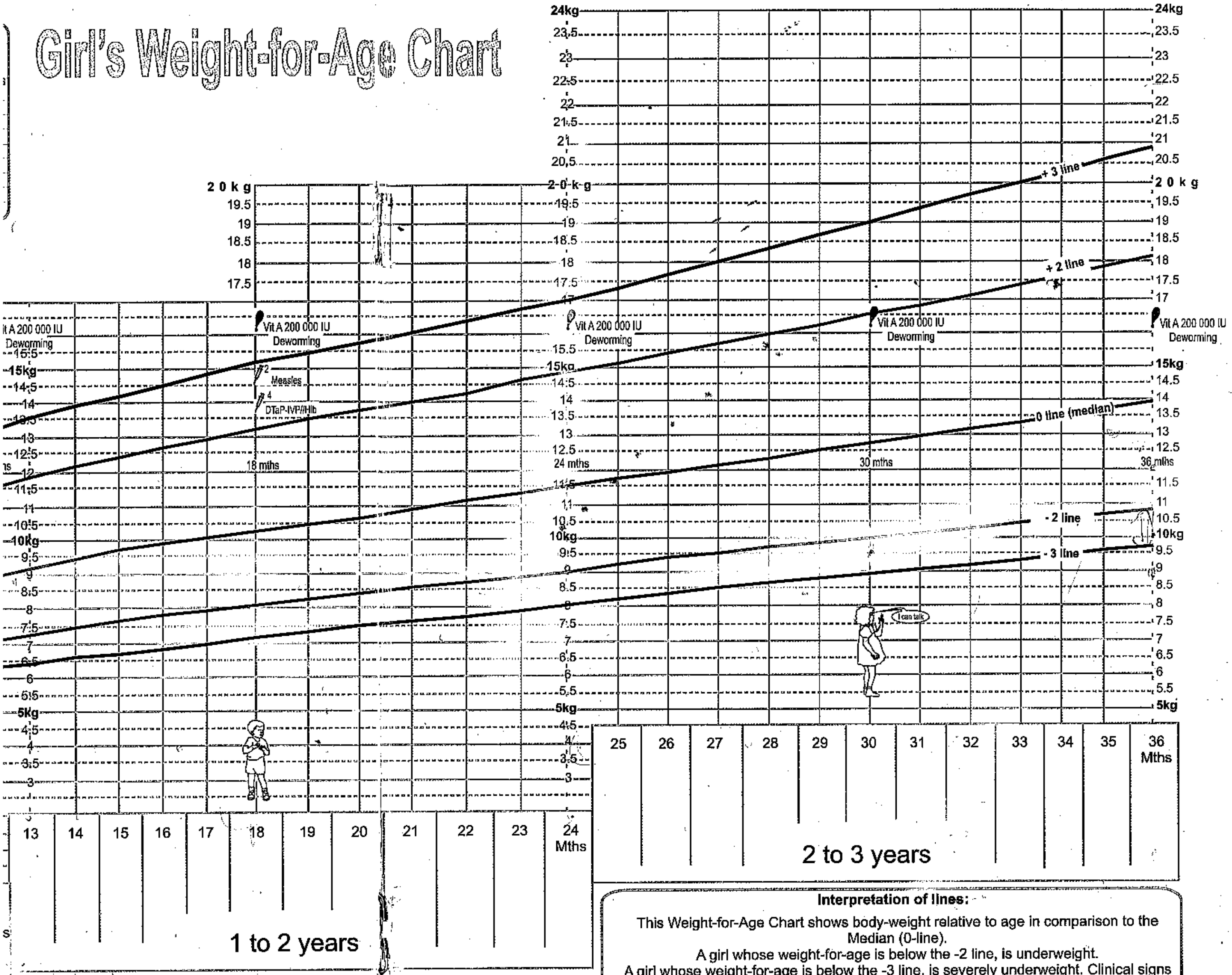

Interpretation of lines:

This Weight-for-Age Chart shows body-weight relative to age in comparison to the Median (0-line).
A girl whose weight-for-age is below the -2 line, is underweight.
A girl whose weight-for-age is below the -3 line, is severely underweight. Clinical signs of Marasmus or Kwashiorkor may be observed.

If her line crosses a z-score line and the shift is away from the median, this may indicate a problem or risk of a problem.

If her line stays close to the median, occasionally crossing above or below it, this is fine.

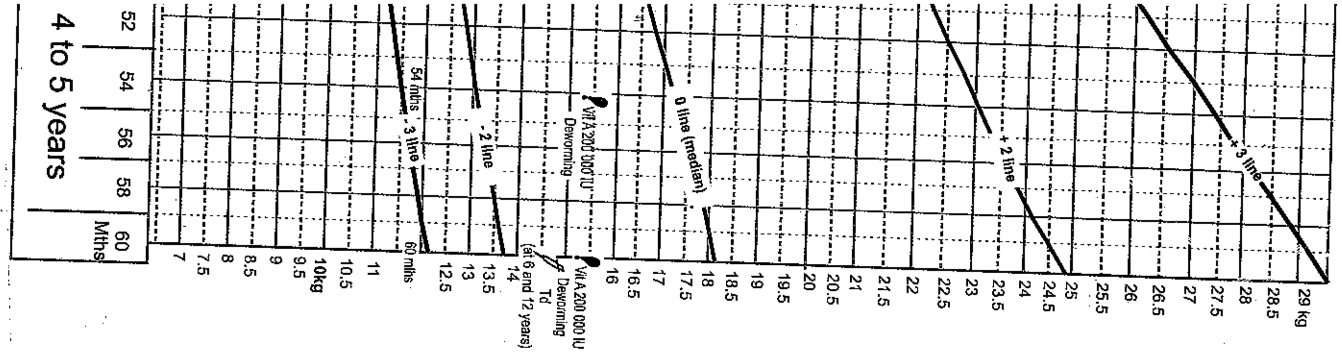

Length/height -for-age GIRLS

Birth to 5 years (z-scores)

FOR PERIODIC USE
(every 6 months) Indicate under "Growth" (page 2 & 3) if child is stunted

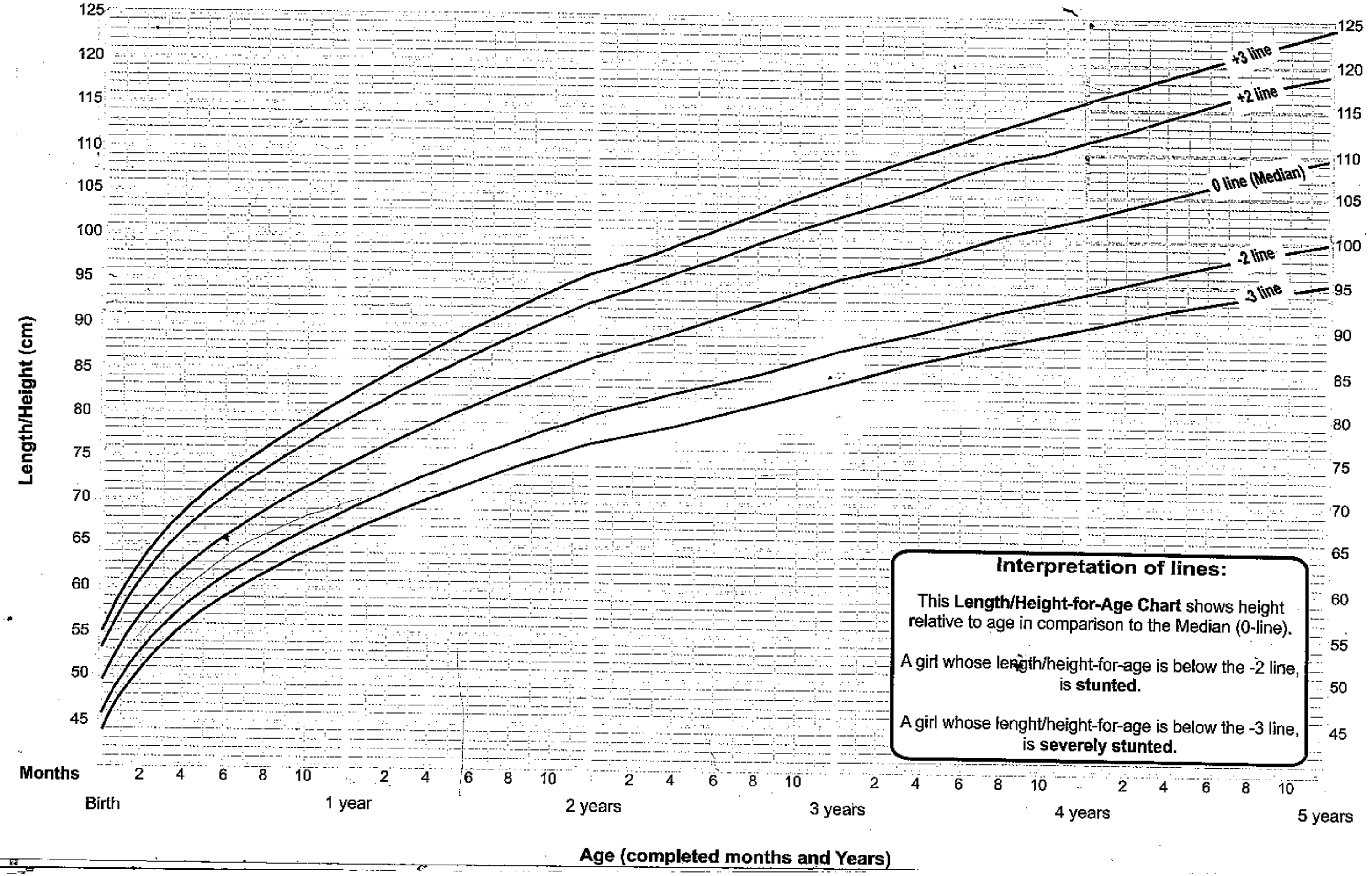

Interpretation of lines:

This **Length/Height-for-Age Chart** shows height relative to age in comparison to the Median (0-line).

A girl whose length/height-for-age is below the -2 line, is **stunted.**

A girl whose lenght/height-for-age is below the -3 line, is **severely stunted.**

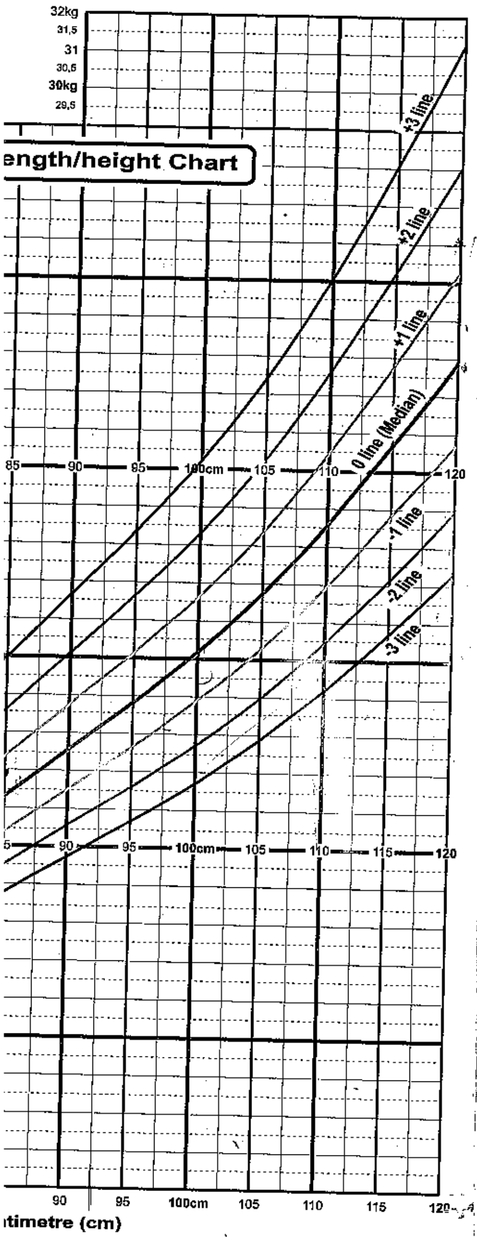

ht relative to length/height in comparison
ore line).

ve the +3 line, is obese.
the +2 line, is overweight.
, shows possible risk of overweight.
w the -2 line, is wasted.
ly wasted. Refer for urgent specialised care.

MID-UPPER ARM CIRCUMFERENCE (MUAC) (Every 3 months)

Date of visit	MUAC	Date of visit	MUAC	Date of visit	MUAC	Date of visit	MUAC

< 11.5 cm indicates severe acute malnutrition (REFER urgently)
≥11.5 < 12.5 cm indicates moderate acute malnutrition (Manage as in IMCI guidelines)

HOSPITAL ADMISSIONS

Hospital name	Admission number	Date of admission dd/mm/yyyy	Date of discharge dd/mm/yyyy	Discharge diagnosis
		/ /	/ /	
		/ /	/ /	
		/ /	/ /	
		/ /	/ /	
		/ /	/ /	
		/ /	/ /	
		/ /	/ /	
		/ /	/ /	
		/ /	/ /	
		/ /	/ /	
		/ /	/ /	
		/ /	/ /	

NAME OF CLINIC(S) VISITED

Clinic 1:	Clinic 2:
Clinic 3:	Clinic 4:

ATIONS

t been done.
ır Oral Hygienist.

Date:

Date:

Date:

Date:

Date:

Date:

CLINICAL NOTES

Date	Assess and classify	Counsel and treat
