# Supplementary material for: Addressing the quality and scope of paediatric primary care in South Africa: evaluating contextual impacts of the introduction of the Practical Approach to Care Kit for children (PACK Child)
Source: BMC Health Serv Res. 2020 May 29;20:479. doi: 10.1186/s12913-020-05201-w (PMC7257217; doi:10.1186/s12913-020-05201-w)
Supplement: Supplementary file 5 — Additional file 5. Observation guide-Non Clinical Areas. Observation guide for non-clinical areas. [file 12913_2020_5201_MOESM5_ESM.pdf]

Additional File 5

Observation Guide-Non-Clinical Areas

V1.0 13/07/2017

PACK CHILD Study
Ethnographic Observations

Observations of Non-Clinical Areas

- What is the layout of the clinic? How is the area you are observing designed?
- Which staff are working? Numbers and different types.
- How are patients accessing care? Are they just turning up?
- What gatekeepers are there? Reception staff? What conversations are patients having with staff in order to access care for their child?
- Are any procedures or protocols being followed?
- How are patients managed when they arrive? Is there any face to face triage?
- Can you track how individual patients are managed and treated? Are they having just one consultation or several?
- Note down any interesting quotes, particularly any points of tension between staff/patients.
