# Supplementary material for: Addressing the quality and scope of paediatric primary care in South Africa: evaluating contextual impacts of the introduction of the Practical Approach to Care Kit for children (PACK Child)
Source: BMC Health Serv Res. 2020 May 29;20:479. doi: 10.1186/s12913-020-05201-w (PMC7257217; doi:10.1186/s12913-020-05201-w)
Supplement: Supplementary file 6 — Additional file 6. PACK Child Training Programme and Cascade Model. Model of Training and Cascade Model. [file 12913_2020_5201_MOESM6_ESM.pdf]

## PACK Child Training Programme and Cascade Model

<table>
<tr><th>Phase</th><th>1</th><th>2</th><th>3</th><th>Training</th></tr>
<tr><td>Master Trainers</td><td colspan="3" rowspan="3">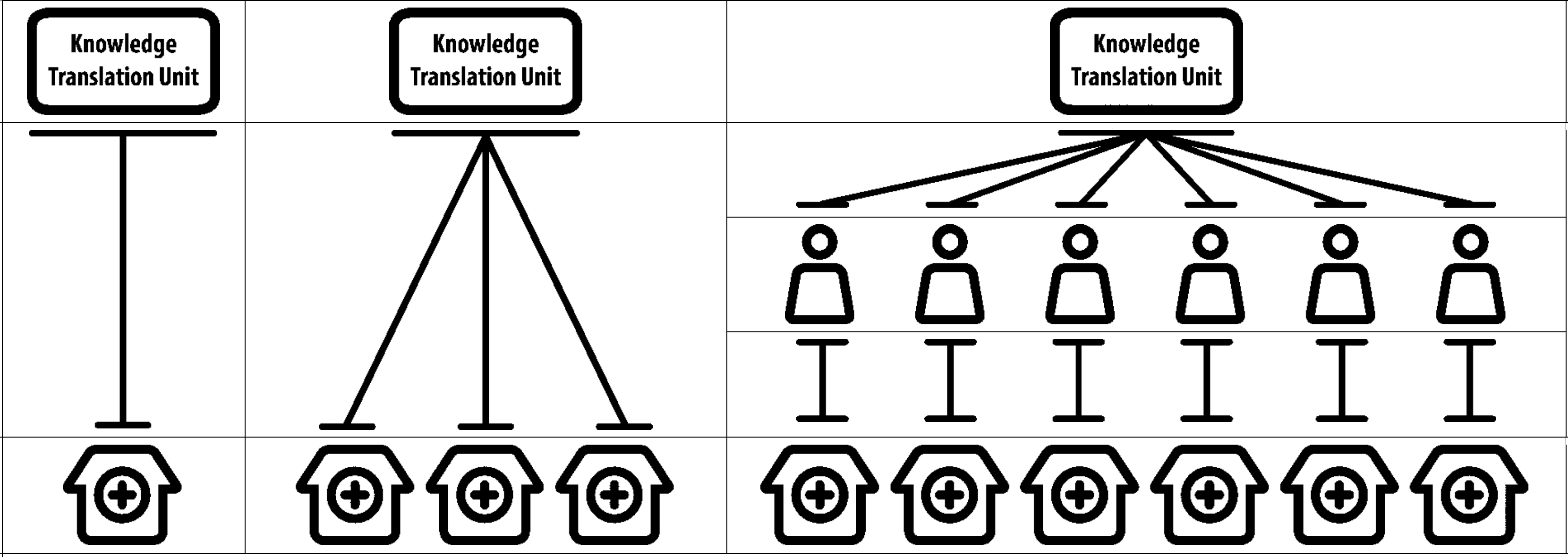
</td><td></td></tr>
<tr><td>Facility Trainers</td><td>Nine sessions delivered over five days</td></tr>
<tr><td>Clinics & staff</td><td>Nine sessions delivered weekly</td></tr>
<tr><td>Summary of training sessions</td><td colspan="4">1. Introductory activity<br>2. Introduce routine care (practise integrating routine care and a symptom)<br>3. Practise integrating routine care & symptom<br>4. Growth activity<br>5. Introduction to the young infant ≤ 2 months (documentation)<br>6. Health system strengthening session<br>7. Diagnosing long term health conditions<br>8. TB<br>9. HIV</td></tr>
</table>
